# Genome-wide identification and characterisation of Aquaporins in *Nicotiana tabacum* and their relationships with other Solanaceae species

**DOI:** 10.1186/s12870-020-02412-5

**Published:** 2020-06-09

**Authors:** Annamaria De Rosa, Alexander Watson-Lazowski, John R. Evans, Michael Groszmann

**Affiliations:** 1 ARC Centre of Excellence for Translational Photosynthesis, Research School of Biology, Australian National University, ACT, Canberra, 2601 Australia; 2 ARC Centre of Excellence for Translational Photosynthesis, Hawkesbury Institute for the Environment, Western Sydney University, Sydney, NSW 2751 Australia

**Keywords:** Aquaporins, Major intrinsic protein, Orthologs, Phylogenetics, Gene structure and evolution, Gene expression profile, *Nicotiana sylvestris*, *Nicotiana tomentosiformis*, *Solanum lycopersicum*, *Solanum tuberosum*

## Abstract

**Background:**

Cellular membranes are dynamic structures, continuously adjusting their composition, allowing plants to respond to developmental signals, stresses, and changing environments. To facilitate transmembrane transport of substrates, plant membranes are embedded with both active and passive transporters. Aquaporins (AQPs) constitute a major family of membrane spanning channel proteins that selectively facilitate the passive bidirectional passage of substrates across biological membranes at an astonishing 10^8^ molecules per second. AQPs are the most diversified in the plant kingdom, comprising of five major subfamilies that differ in temporal and spatial gene expression, subcellular protein localisation, substrate specificity, and post-translational regulatory mechanisms; collectively providing a dynamic transportation network spanning the entire plant. Plant AQPs can transport a range of solutes essential for numerous plant processes including, water relations, growth and development, stress responses, root nutrient uptake, and photosynthesis. The ability to manipulate AQPs towards improving plant productivity, is reliant on expanding our insight into the diversity and functional roles of AQPs.

**Results:**

We characterised the AQP family from *Nicotiana tabacum* (NtAQPs; tobacco), a popular model system capable of scaling from the laboratory to the field. Tobacco is closely related to major economic crops (e.g. tomato, potato, eggplant and peppers) and itself has new commercial applications. Tobacco harbours 76 AQPs making it the second largest characterised AQP family. These fall into five distinct subfamilies, for which we characterised phylogenetic relationships, gene structures, protein sequences, selectivity filter compositions, sub-cellular localisation, and tissue-specific expression. We also identified the AQPs from tobacco’s parental genomes (*N. sylvestris* and *N. tomentosiformis*), allowing us to characterise the evolutionary history of the NtAQP family. Assigning orthology to tomato and potato AQPs allowed for cross-species comparisons of conservation in protein structures, gene expression, and potential physiological roles.

**Conclusions:**

This study provides a comprehensive characterisation of the tobacco AQP family, and strengthens the current knowledge of AQP biology. The refined gene/protein models, tissue-specific expression analysis, and cross-species comparisons, provide valuable insight into the evolutionary history and likely physiological roles of NtAQPs and their Solanaceae orthologs. Collectively, these results will support future functional studies and help transfer basic research to applied agriculture.

## Background

Cellular membranes are dynamic structures, continuously adjusting their composition in order to allow plants to respond to developmental signals, stresses, and changing environments [1]. The biological function of cell membranes is conferred by its protein composition, with the lipid bilayer providing a basic structure and permeability barrier, and integral transmembrane proteins facilitating diffusion of selected substrates [1]. Cell membrane diffusion is a fundamental process of plant biology and one of the oldest subjects studied in plant physiology [2]. Diffusional events at the cellular level eventuate in the coordinated transport of substrates throughout the plant to support development and growth.

Plant membranes contain three major classes of transport proteins known as ATP-powered pumps, Transporters, and Channel proteins [3]. Pumps, are active transporters that use the energy of ATP hydrolysis to move substrates across the membrane against a concentration gradient or electrical potential. Transporters move a variety of molecules across a membrane along or against a gradient at rates of 10^2^ to 10^4^ molecules per second. Unlike the first two classes, channel proteins are bidirectional and increase membrane permeability to a particular molecule. Channel proteins are permeable to a wide range of substrates and can pass up to 10^8^ molecules per second. In plants, aquaporins (AQPs) constitute a major family of such channel proteins that facilitate selective transport of substrates for numerous biological processes including, water relations, plant development, stress responses, and photosynthesis [4, 5].

The AQP monomer forms a characteristic hour-glass membrane-spanning pore that assembles as tetrameric complexes in cell membranes. The union of the four monomers, creates a fifth pore at the centre of the tetramer which may provide an additional diffusional path [6]. The substrate specificity of a given AQP is conferred by the complement of pore lining residues which achieve specificity through a combination of size exclusion and biochemical interactions with substrates [7]. Key identified specificity residues include the dual Asn-Pro-Ala (NPA) motifs, the aromatic/Arginine filter (ar/R filter) and Froger’s positions (P1-P5) [8–10]. However, other pore-lining residues and lengths of the various transmembrane and loop domains of the AQP monomer are also known to influence substrate specificity through conformational changes of the pore size and accessibility [7, 11]. It is likely that other residues that determine specificity and transport efficiency remain to be elucidated.

Aquaporins, which are members of the major intrinsic proteins (MIP) superfamily, are found across all taxonomic kingdoms [12]. While mammals usually have only 15 isoforms, plants have vastly larger AQP families commonly ranging from 30 to 121 members [5, 13–15]. This impressive diversification has been facilitated by the propensity of gene duplication events, especially prevalent in the angiosperms, and likely by the adaptive potential provided by AQPs. Based on sequence homology and subcellular localisation, up to thirteen AQP subfamilies are now recognised in the plant kingdom [13, 16–19]. Eight of these AQP subfamilies occur in more ancestral plant lineages and include, the GlpF-like Intrinsic Proteins (GIPs) and Hybrid Intrinsic Proteins (HIPs) in mosses, the MIPs A to E of green algae, and the Large Intrinsic Proteins (LIPs) in diatoms. The remaining five subfamilies are prevalent across higher plants and have extensively diversified into sub-groups and include the Plasma membrane Intrinsic Proteins (PIPs; subgroups PIP1 and PIP2), Tonoplast Intrinsic Proteins (TIPs; subgroups TIP1 to TIP5), Small basic Intrinsic Proteins (SIPs; subgroups SIP1 and SIP2), Nodulin 26-like Intrinsic Proteins (NIPs; subgroups NIP1 to NIP5), and X Intrinsic Proteins (XIPs; subgroups XIP1 to XIP3). The XIPs are present in many eudicot species, but are absent in the Brassicaceae and monocots [17].

The AQP subfamilies differ to some degree in substrate specificity and integrate into different cellular membranes, providing plants with a versatile system for both sub-cellular compartmentalisation and intercellular transport. In plants, AQPs are by far the most extensively diversified, capable of transporting a wide variety of substrates including water, ammonia, urea, carbon dioxide, hydrogen peroxide, boron, silicon and other metalloids [7, 20, 21]. More recently, lactic acid, oxygen, and cations have been identified as permeating substrates [22–25], with RNA molecules also implicated as a possible transported substrate [26]. Further versatility is achieved through tightly regulated spatial and temporal tissue-specific expression of different *AQP* genes, as well as post-translational modification of AQP proteins (e.g. phosphorylation) that controls membrane trafficking and channel activity [27, 28].

Given their diverse complement of transported substrates and growing involvement in many developmental and stress responsive physiological roles, AQPs are targets for engineering more resilient and productive plants [5, 29]. For example, CO_2_-permeable AQP are being targeted to enhance photosynthetic efficiency and yield increases [5, 30, 31], while AQPs responsive to drought stress are being used to improve tolerance to water-limited conditions [32, 33], and manipulations of boron-permeable AQPs are being pursued to improve crop tolerance to soils with either toxic or sub-optimal levels of boron [15, 34, 35]. The genomic era of plant biology has provided unprecedented opportunity to query AQP biology by exploring sequence conservation and diversity between isoforms in many species. This is reflected in the increasing number of plant AQP family studies being reported in recent years. Almost exclusively, these studies focus on the species of interest with no direct evaluation with AQPs from other plant species. However, extending an AQP family characterisation to closely related species (e.g. within the same taxonomic family) can be especially informative, with comparisons of close orthologous AQPs helping to better elucidate the evolutionary history and physiological roles of different AQPs. Comparisons between closely related species can also improve the translation of basic AQP research to applied agriculture, especially if the analysis involves crop species.

To improve our current knowledge on AQP biology and aid in their potential use towards improving plant resilience and productivity, we have characterised the AQP family from *Nicotiana tabacum* (NtAQPs; tobacco). Tobacco is a fitting candidate species to explore unknowns of AQP biology as it is a popular model system for studying fundamental physiological processes that is capable of scaling from the laboratory to the field. Tobacco is part of the large Solanaceae family, which includes species of major economic importance such as tomato, potato, eggplant and peppers [36], and itself has renewed commercial applications in the biofuel and plant-based pharmaceutical sectors [37–39]. We found that tobacco harbours 76 AQPs, making it the second largest family characterised to date. Tobacco is a recent allotetraploid, which accounts for its large AQP family size. Phylogenetic relationships, gene structures, protein sequences, selectivity filter compositions, sub-cellular localisation, and tissue-specific expression profiles were used to characterise NtAQP family members. We also identified the AQPs of the tobacco parental genomes (*Nicotiana sylvestris* and *Nicotiana tomentosiformis*), allowing us to characterise the recent evolutionary history of the NtAQP family. Furthermore, using the already defined AQP families of tomato (*Solanum lycopersicum*) and potato (*Solanum tuberosum*) [40, 41], we made cross-species comparisons of gene structures, protein sequences and expression profiles, to provide insight into conservation and diversification of protein function and physiological roles for future studies and engineering efforts.

## Results

### Identification and classification of NtAQP genes

**Fig. 1:**
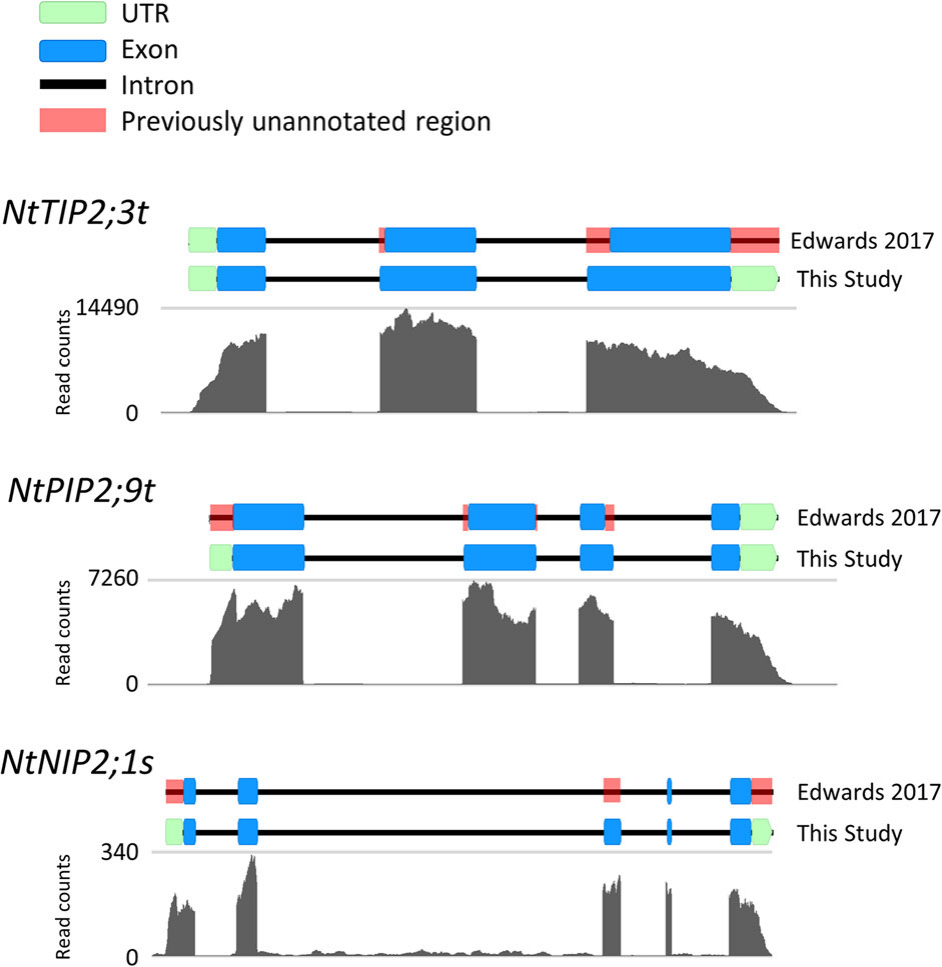
Representative examples of our curated gene models validated with RNA-seq data. Our curated models were aligned to those computed in Edwards et al. (2017). The examples depicted in the figure have high (*NtTIP2;3 t*), medium (*NtPIP2;9 t*) and low expression levels (*NtNIP2;1 s*). Mapped genomic reads locate to mRNA encoding regions and as such denote exon boundaries and UTRs. Red boxes in the Edwards predicted gene models denote missing coding regions as indicated by deviations from the RNA-seq localisation

A homology search, using tomato and potato AQP protein sequences as queries, identified 85 loci putatively encoding AQP-like genes in the genome of the TN90 tobacco cultivar [42]. Nine of these genes encode for severely truncated proteins and were classified as pseudogenes (Additional file 1: Table S1). The remaining 76 genes had a level of homology to tomato and potato AQPs to be considered ‘bona fide’ tobacco AQPs (NtAQPs; Table 1). Seventy-three of these 76 tobacco AQP genes were also identified in the genome of the more recently sequenced K326 cultivar (Nitab4.5v) [43] (Table 1). To determine the precise protein sequences and gene structures of the tobacco AQPs, the surrounding genomic region of the identified coding sequences were examined in all forward translated frames. The likely protein products and associated intron/exon structures were curated through alignments with respective Solanaceae homologues. Our gene models were then independently validated and supported by alignments against tobacco whole transcriptome mRNA-seq data (obtained from Edwards et al., 2017), which also aided in defining the 5′ and 3′ UTRs. A comparison between our manually curated AQP protein and gene models against the computational predictions for the TN90 and K326 cultivars [42, 43] revealed that 15% of TN90 and 50% of K326 computed AQP models were incorrectly annotated (Table 1). Errors in the computed gene models were encountered across all NtAQP subfamilies and consisted of either missing or truncated 5′ and 3’UTRs, absent exons, truncated exons (ranging from 4 to 87 amino acids), and exon insertions (16–57 amino acids) due to inclusion of adjacent intron sequence (Fig. 1, Additional file 2: Figure S1). A summary of our NtAQP gene models, identifiers and genomic locations for the TN90 and K326 cultivars are available in Additional file 1: Table S2. FASTA sequencing files of coding DNA sequence (CDS), protein, and genomic sequence can be found in Additional file 3. Sequences of these high confidence NtAQP protein and gene models have been submitted to NCBI (Table 1).

**Table 1:** List of the 76 tobacco aquaporin genes identified in this study

This study			TN90 - Sierro et al., 2014		K326 - Edwards et al., 2017	
Gene ID	Protein (aa)	NCBI accession - This study	Gene ID^(1)^	Accurate gene model?^(1)^	Gene ID^(2)^	Accurate gene model?^(2)^
NtPIP1;1s	289	BK011392	gene_35182	Y	Nitab4.5_0004836g0030.1	N
NtPIP1;1t	289	BK011393	gene_27714	Y	Nitab4.5_0006090g0020.1	N
NtPIP1;2s	288	BK011394	gene_58674	Y	Nitab4.5_0011459g0010.1	Y
NtPIP1;2t	286	BK011395	gene_10991	N	Nitab4.5_0000583g0150.1	Y
NtPIP1;3s	288	BK011396	gene_79275	Y	Nitab4.5_0007597g0010.1	Y
NtPIP1;3t	288	BK011397	gene_84661	Y	Nitab4.5_0003043g0010.1	Y
NtPIP1;5s	288	BK011398	gene_40739	Y	Nitab4.5_0010813g0020.1	N
NtPIP1;5t	288	BK011399	gene_80239	Y	Nitab4.5_0001615g0140.1	Y
NtPIP1;7s	287	BK011400	gene_59749	Y	Nitab4.5_0006718g0030.1	N
NtPIP1;8s	286	BK011401	gene_86041	Y	Nitab4.5_0000737g0120.1	Y
NtPIP2;1s	284	BK011402	gene_9798	Y	Nitab4.5_0009795g0020.1	Y
NtPIP2;1x	284	BK011403	gene_9795	N	Nitab4.5_0009795g0010.1	N
NtPIP2;2t	284	BK011404	gene_87071	Y	Nitab4.5_0000101g0110.1	Y
NtPIP2;3t	284	BK011405	gene_8898	N	Nitab4.5_0000101g0120.1	N
NtPIP2;4s	288	BK011406	gene_84258	Y	Nitab4.5_0004314g0010.1	Y
NtPIP2;4t	288	BK011407	gene_71307	Y	Nitab4.5_0000181g0120.1	N
NtPIP2;5s	286	BK011408	gene_31592	Y	Nitab4.5_0001192g0080.1	N
NtPIP2;5t	286	BK011409	gene_32945	Y	Nitab4.5_0001297g0050.1	N
NtPIP2;6s	288	BK011410	gene_22735	Y	Nitab4.5_0004108g0020.1	Y
NtPIP2;6t	288	BK011411	gene_34319	Y	Nitab4.5_0000650g0260.1	Y
NtPIP2;7t	284	BK011412	gene_84225	Y	Nitab4.5_0000106g0170.1	Y
NtPIP2;8s	285	BK011413	gene_75147	Y	Nitab4.5_0003914g0040.1	Y
NtPIP2;8t	285	BK011414	gene_53392	Y	Nitab4.5_0000283g0420.1	Y
NtPIP2;9s	284	BK011415	gene_84936	Y	Nitab4.5_0005236g0020.1	N
NtPIP2;9t	284	BK011416	gene_9787	N	Nitab4.5_0002763g0030.1	N
NtPIP2;11s	269	BK011417	gene_40272	Y	Nitab4.5_0008552g0040.1	N
NtPIP2;11t	269	BK011418	gene_62966	Y	Nitab4.5_0001789g0070.1	N
NtPIP2;13s	284	BK011419	gene_55607	Y	Nitab4.5_0014443g0010.1	Y
NtPIP2;13t	284	BK011420	gene_81728	Y	Nitab4.5_0000575g0130.1	Y
NtNIP1;1s	275	BK011376	gene_27146	N	Nitab4.5_0005428g0060.1	N
NtNIP1;2s	288	BK011377	gene_42864	Y	Nitab4.5_0008572g0060.1	N
NtNIP1;2t	282	BK011378	gene_42851	N	Nitab4.5_0001778g0110.1	Y
NtNIP2;1s	287	BK011379	gene_24518	Y	Nitab4.5_0001638g0020.1	N
NtNIP3;1s	348	BK011380	gene_85282	Y	Nitab4.5_0013395g0010.1	N
NtNIP4;1s	271	BK011381	gene_11802	Y	Nitab4.5_0003360g0080.1	N
NtNIP4;1t	272	BK011382	gene_33173	Y	Nitab4.5_0004399g0020.1	N
NtNIP4;2s	273	BK011383	gene_47152	Y	Not identified	-
NtNIP4;2t	273	BK011384	gene_36231	Y	Nitab4.5_0000742g0130.1	N
NtNIP4;3s	282	BK011385	gene_55126	Y	Not identified	-
NtNIP5;1s	298	BK011386	gene_36225	Y	Nitab4.5_0005519g0010.1	N
NtNIP5;1t	298	BK011387	gene_38118	Y	Nitab4.5_0000799g0080.1	Y
NtNIP6;1s	304	BK011388	gene_39457	N	Nitab4.5_0012943g0030.1	Y
NtNIP6;1t	304	BK011389	gene_8958	N	Nitab4.5_0001454g0120.1	N
NtNIP7;1s	294	BK011390	gene_69139	Y	Nitab4.5_0007039g0010.1	N
NtNIP7;1t	281	BK011391	gene_41519	Y	Nitab4.5_0002600g0020.1	N
NtTIP1;1s	252	BK011426	gene_4702	Y	Nitab4.5_0003155g0010.1	N
NtTIP1;1t	252	BK011427	gene_17915	Y	Nitab4.5_0001163g0070.1	N
NtTIP1;2s	253	BK011428	gene_62289	Y	Nitab4.5_0001068g0010.1	Y
NtTIP1;2t	253	BK011429	gene_18091	Y	Nitab4.5_0000766g0050.1	Y
NtTIP1;3t	249	BK011430	gene_34364	Y	Nitab4.5_0022765g0010.1	Y
NtTIP1;3s	249	BK011431	gene_81216	Y	Nitab4.5_0011193g0010.1	Y
NtTIP1;4t	252	BK011432	gene_44062	Y	Nitab4.5_0000173g0030.1	N
NtTIP2;1s	249	BK011433	gene_13886	N	Nitab4.5_0009267g0020.1	N
NtTIP2;1t	249	BK011434	gene_84779	Y	Nitab4.5_0003039g0050.1	N
NtTIP2;2s	251	BK011435	gene_65205	Y	Nitab4.5_0001381g0190.1	N
NtTIP2;3s	251	BK011436	gene_8782	Y	Nitab4.5_0001076g0030.1	N
NtTIP2;3t	251	BK011437	gene_77281	Y	Nitab4.5_0000618g0070.1	N
NtTIP2;4s	249	BK011438	gene_44575	Y	Nitab4.5_0007573g0030.1	Y
NtTIP2;5s	249	BK011439	gene_55803	Y	Not identified	-
NtTIP2;5t	249	BK011440	gene_36783	Y	Nitab4.5_0011578g0040.1	N
NtTIP3;1s	260	BK011441	gene_7183	Y	Nitab4.5_0005315g0010.1	Y
NtTIP3;1t	260	BK011442	gene_54243	Y	Nitab4.5_0000477g0090.1	Y
NtTIP3;2t	259	BK011443	gene_79868	N	Nitab4.5_0009307g0020.1	Y
NtTIP4;1s	248	BK011444	gene_76645	Y	Nitab4.5_0000837g0080.1	N
NtTIP4;1t	248	BK011445	gene_2305	Y	Nitab4.5_0000151g0360.1	Y
NtTIP5;1s	251	BK011446	gene_8008	Y	Nitab4.5_0010023g0020.1	Y
NtTIP5;1t	251	BK011447	gene_33209	Y	Nitab4.5_0002816g0050.1	N
NtSIP1;1t	238	BK011421	gene_54009	N	Nitab4.5_0000001g0350.1	Y
NtSIP1;2s	244	BK011422	gene_73217	Y	Nitab4.5_0007223g0030.1	Y
NtSIP1;2t	243	BK011423	gene_74850	Y	Nitab4.5_0000812g0160.1	N
NtSIP2;1s	241	BK011424	gene_42066	Y	Nitab4.5_0001918g0070.1	N
NtSIP2;1t	241	BK011425	gene_29131	N	Nitab4.5_0000721g0170.1	Y
NtXIP1;6s	327	BK011448	gene_13292	Y	Nitab4.5_0007293g0050.1	N
NtXIP1;6t	327	BK011449	gene_52652	Y	Nitab4.5_0000956g0150.1	N
NtXIP1;7s	314	BK011450	gene_34706	Y	Nitab4.5_0007733g0020.1	N
NtXIP1;7t	314	BK011451	gene_50247	Y	Nitab4.5_0006828g0010.1	N

List of the 76 tobacco aquaporin genes identified in this study. Provided are protein lengths, gene identifiers in the TN90(1) (Sierro et al. 2014) and K326(2) (Edwards et al. 2017) cultivar genomes, comparison of whether the computational gene models derived from each study matched the curated gene structures (Y-yes or N-no) and NCBI accession identifiers. NtTIP2;5s, NtNIP4;2s and NtNIP4;3t genes were not identified in the K326(2) cultivar’s genome. For further details, including gene identifiers, previous NCBI accession identifiers etc.., see the expanded version of this table in Additional File 1: Table S2

Through the process of curating the tobacco AQP gene and protein sequences, we have made correction to several previously mis-annotated AQP genes of tomato and potato namely, *StXIP3;1, StXIP4;1*, *SlXIP1;6*, *SlPIP2;1*, and *SlTIP2;2* (Additional file 1; Table S3). We also identified through our tobacco genome sequence analysis an erroneous non-synonymous single nucleotide mutation (C > T, CDS position 619) in the reported mRNA sequence of the frequently studied tobacco AQP1 gene (NtAQP1; assigned as NtPIP1;5 s in this study). The mutation results in a Histidine (H) to Tyrosine (Y) substitution at amino acid position 207 being incorrectly reported in the initial cloning of this gene and subsequent use ( [44]; NCBI AF024511 and AJ001416). This substitution is notable since His207, which corresponds to the His193 position of the well-studied crystal structures of Spinach PIP2;1 [6, 45, 46], is highly conserved across all angiosperm PIP AQPs and is a key regulator in the gating and therefore transport capacity of the AQP channel [6, 45, 47]. The inadvertent use of this H207Y NtAQP1 mutant in functional characterisation studies may have implication on the conclusions drawn for this frequently studied plant AQP. In support of His207 being the correct residue in NtAQP1, we found that independently generated gDNA-seq assemblies as well as RNA-seq mapped reads from both the TN90 and K326 cultivars had the His207 residue (Additional file 2: Figure S2). Furthermore, several closely related NtAQP1 orthologues across several Solanaceae species, including 3 additional *Nicotiana* species, all had the His207 residue (Additional file 2: Figure S2).

### Gene structures and phylogenetic analysis of tobacco AQPs

**Fig. 2:**
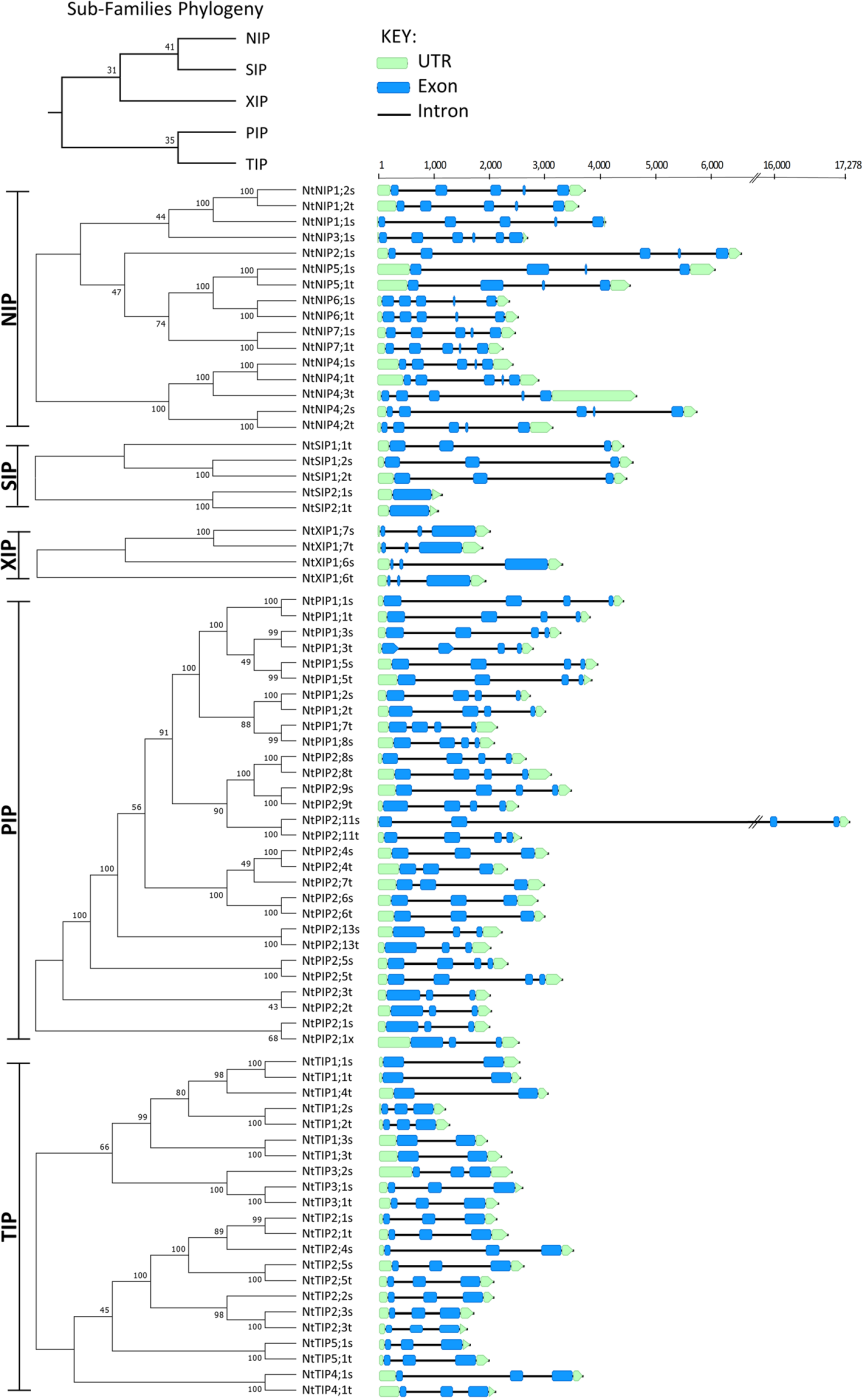
Phylogeny and gene structures of 76 tobacco aquaporins. Phylogenetic tree was generated using the neighbour-joining method (via MEGA7) from MUSCLE aligned protein sequences. Confidence levels (%) of branch points generated through bootstrapping analysis (*n* = 1000). Gene structures are located adjacent to their respective location on the phylogenetic tree; blue rectangles correspond to the exons; green rectangles and arrows to the 5′ and 3’UTRs, respectively. Scale bar at the top of gene structures indicates nucleotide length. The last letter in the NtAQP names denote the likely origin of the gene (s = *N. sylvestris*, t = *N. tomentosiformis*, x = unknown)

To place the 76 curated NtAQP protein sequences into their respective subfamilies, we used phylogenetic analyses incorporating characterised AQP isoforms from a diverse set of angiosperms: Arabidopsis (*Arabidopsis thaliana*, Brassicales), tomato (*Solanum lycopersicum,* Solanales), rubber tree (*Hevea brasiliensis,* Malpighiales), rice (*Oryza sativa,* Poales) and soy bean (*Glycine max*, Fabales) (Additional file 4: Figure S3). The NtAQPs segregated into five distinct subfamilies that commonly occur in higher plants, namely the NIPs [16], SIPs [5], XIPs [4], PIPs [29] and TIPs [22] (Fig. 2, Additional file 4: Figure S3). An emerging problem among the increasing number of studies characterising plant AQP families across species is the confusion in nomenclature that either misses or incorrectly assigns orthology between AQP genes. Such confusion is seen in the nomenclature between tomato and potato AQPs. At least in this case, the naming inconsistency is predominantly a result of the two family characterisations being published concurrently by different groups [40, 41]. Towards contributing to a more congruent naming structure of AQPs between species, especially within a single family of angiosperms, we aligned our NtAQP naming convention with that of tomato AQPs, given their more consistent nomenclature to likely Arabidopsis AQP orthologues. Additional file 1: Table S2 lists the tobacco AQPs with their corresponding tomato and potato orthologous genes.

Sixty five of the 76 NtAQP genes had clear orthologs in tomato which directed their naming (Additional file 2: Figure S4 and Additional file 1: Table S2). The 11 tobacco *AQPs* with no apparent tomato or potato ortholog were allocated designations unique to tobacco (denoted by black stars in Additional file 2: Figure S4). Gene lengths varied between NtAQPs from 1091 bp to 6627 bp, with a single extreme instance of 17,278 bp (*NtPIP2;11 s*) due to a large intron insertion (Fig. 2). The exon-intron patterning of NtAQP genes were highly conserved with that of their tomato and potato orthologs (Additional file 1: Table S2) [40, 41]. Individual AQPs within the PIP, TIP, NIP and SIP subfamilies were well conserved across the three Solanaceae species (Additional file 2: Figure S4). The XIPs were an exception as they predominantly phylogenetically clustered within each separate species, pointing to a high degree of intra-species XIP diversification within the Solanaceae (Additional file 2: Figure S4).

A distinctive feature in the phylogeny was that most NtAQPs reside as pairs, supported by high bootstrap values (Fig. 2). The high homology in protein sequences between members of these phylogenetic pairs also extended to highly similar nucleotide sequences and gene structures (Fig. 2).

### Tobacco AQP protein sequence comparisons

#### General structural features of NtAQP proteins

**Fig. 3:**
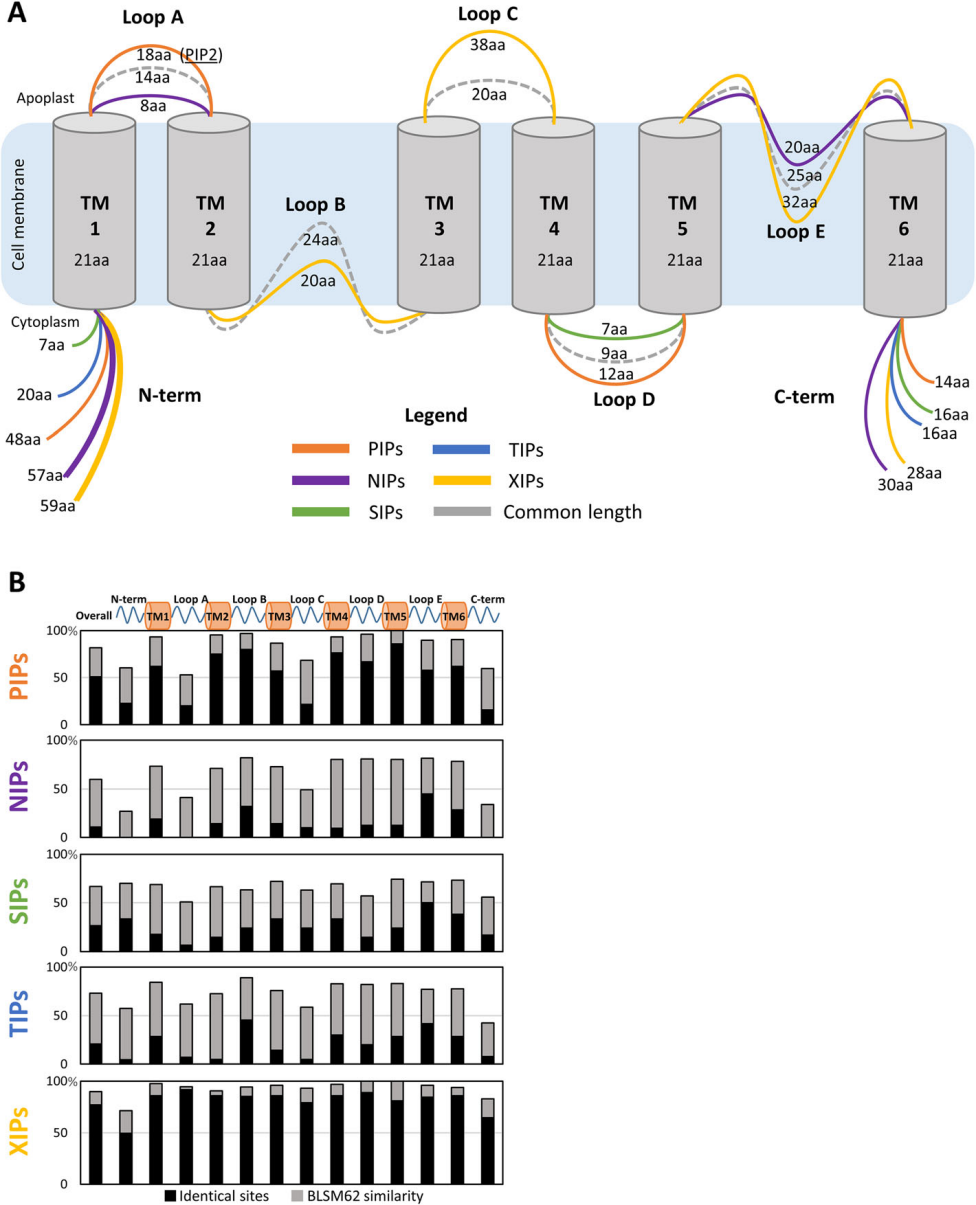
Protein sequence comparisons of NtAQP subfamilies. **a** Diagrammatic illustration of an AQP depicting protein topology and lengths of the; N-terminal tail (N-term), TransMembrane domains (TM) 1–6, Loops A-E, and C-terminal tail (C-term). The average amino acid (aa) lengths of each structural feature are listed for the different NtAQP subfamilies. Common length of a domain is represented in grey, while deviations from the common length are in colour; PIPs (orange), NIPs (purple), SIPs (green), TIPs (blue) and XIPs (yellow). **b** Overall and intra-domain sequence similarities for each NtAQP sub-family. Schematic representation of the AQP domains is illustrated at the top, with aligned columns showing protein sequence identical sites (black) and the BLSM62 similarity score (grey) between members of the given NtAQP subfamily

Topological analysis using TOPCON (see materials and methods), predicted that all NtAQP proteins consist of six transmembrane helical domains, five intervening loop regions and cytoplasmic localised N- and C- terminal tails, which is consistent with the typical structure of AQPs (Fig. 3). The size of the transmembrane helical domains appear to be an integral property of the AQP structure given their remarkably conserved lengths across the subfamilies (Fig. 3a). Conversely, the length of the loop regions showed substantial variability between subfamilies (Fig. 3a). The most pronounced was Loop A, which is prominently longer and apoplastically exposed in the PIP2s (18aa) and shorter in the NIPs (8aa) compared to the average length of TIPs, SIPs, and XIPs (14aa). The cytoplasmic Loop B, is shorter in XIPs (20aa vs. 24aa). Loop C is nearly double the length in the XIPs (38aa) compared to the other subfamilies (20aa). Loop D is slightly longer in the PIPs (12aa) and shorter in the SIPs (7aa), while Loop E is substantially longer in the XIPs (32aa) and shorter in the NIPs (20aa) (Fig. 3a). The cytoplasmically localised N- and C-terminal tails are the most varied in size of any of the AQP domains (Fig. 3a). The N-terminal tail ranges from 59aa in the NIPs to just 7aa in the SIPs and the C-terminal tail from 30aa in the NIPs to 14aa in the PIPs.

Examining sequence conservation of the different protein domains across the subfamilies, revealed that the transmembrane helices are generally the more highly conserved feature of the AQP (Fig. 3b). Loop B and E are also highly conserved relative to the other domains, which is likely owing to their direct role in forming the transmembrane pore. Conversely, Loops A and C, along with the two terminal tails were found to be the least conserved domains within each NtAQP sub-family (Fig. 3b).

To learn more about the putative functional characteristics of the different NtAQPs, we used multiple protein sequence alignments to report residue compositions at key positions in the protein known to regulate AQP function (Table 2). Included are the dual Asn-Pro-Ala (NPA) motifs, the five Froger’s position residues (P1-P5), and the residues of the aromatic/Arginine filter (ar/R filter), all of which are specific pore lining residues that contribute to determining which substrates permeate though the AQP pore. We also reported on several other sites known to be post-translationally modified, which influence channel activity and membrane localisation (Table 2).

**Table 2:** Amino acid composition of NtAQPs at known functionally important positions

	Gene Name	NPA I	NPA II	ar/R					Froger's positions					Other regulatory residues							Pred. subcellular loc.		
		LB	LE	H2	LC	H5	LE1	LE2	P1	P2	P3	P4	P5	Asp28	Glu31	Ser115	Ser188	His193	Ser274	Ser277	mPLoc	WolfPsort	Yloc
**PIP**	NtPIP1;1s	**NPA**	**NPA**	**F**	**G**	**H**	**T**	**R**	**M**	**S**	**A**	**F**	**W**	**E**	**E**	**S**^**P**^	**N**	**H**	-	**S**	plas	plas	cyto
	NtPIP1;1t	NPA	NPA	F	G	H	T	R	M	S	A	F	W	E	E	S^P^	N	H	-	S	plas	plas	cyto
	NtPIP1;2s	NPA	NPA	F	G	H	T	R	**Q**	S	A	F	W	E	E	S^P^	N	H	**-**	S	plas	plas	cyto
	NtPIP1;2t	NPA	NPA	F	G	H	T	R	Q	S	A	F	W	E	E	S^P^	N	H	-	S	plas	plas	cyto
	NtPIP1;3s	NPA	NPA	F	G	H	T	R	**M**	S	A	F	W	E	E	S^P^	N	H	-	S	plas	plas	cyto
	NtPIP1;3t	NPA	NPA	F	G	H	T	R	M	S	A	F	W	E	E	S^P^	N	H	-	S	plas	plas	cyto
	NtPIP1;5s	NPA	NPA	F	G	H	T	R	M	S	A	F	W	E	E	S^P^	N	H	-	S	plas	plas	cyto
	NtPIP1;5t	NPA	NPA	F	G	H	T	R	M	S	A	F	W	E	E	S^P^	N	H	-	S	plas	plas	cyto
	NtPIP1;7t	NPA	NPA	F	G	H	T	R	**G**	S	A	F	W	E	E	S^P^	**S**^**P**^	H	-	S	plas	plas	cyto
	NtPIP1;8s	NPA	NPA	F	G	H	T	R	G	S	A	F	W	E	E	S^P^	S^P^	H	-	S	plas	plas	plas
	NtPIP2;1s	NPA	NPA	F	G	H	T	R	**Q**	S	A	F	W	**D**	E	S^P^	**N**	H	**S**^**P**^	S	plas	plas	plas
	NtPIP2;1x	NPA	NPA	F	G	H	T	R	Q	S	A	F	W	D	E	S^P^	N	H	S^P^	S	plas	plas	plas
	NtPIP2;2t	NPA	NPA	F	G	H	T	R	Q	S	A	F	W	D	E	S^P^	N	H	S^P^	S	plas	plas	plas
	NtPIP2;3t	NPA	NPA	F	G	H	T	R	Q	S	A	F	W	D	E	S^P^	N	H	S^P^	S	plas	plas	plas
	NtPIP2;4s	NPA	NPA	F	G	H	T	R	Q	S	A	F	W	D	E	S^P^	N	H	S^P^	S	plas	plas	plas
	NtPIP2;4t	NPA	NPA	F	G	H	T	R	Q	S	A	F	W	D	E	S^P^	N	H	S^P^	S	plas	plas	plas
	NtPIP2;5s	NPA	NPA	F	G	H	T	R	Q	S	A	F	W	D	E	S^P^	N	H	S^P^	S	plas	plas	plas
	NtPIP2;5t	NPA	NPA	F	G	H	T	R	Q	S	A	F	W	D	E	S^P^	N	H	S^P^	S	plas	plas	plas
	NtPIP2;6s	NPA	NPA	F	G	H	T	R	Q	S	A	F	W	D	E	S^P^	**S**^**P**^	H	S^P^	S	plas	plas	plas
	NtPIP2;6t	NPA	NPA	F	G	H	T	R	Q	S	A	F	W	D	E	S^P^	S^P^	H	S^P^	S	plas	plas	plas
	NtPIP2;7t	NPA	NPA	F	G	H	T	R	Q	S	A	F	W	D	E	S^P^	S^P^	H	S^P^	S	plas	plas	plas
	NtPIP2;8s	NPA	NPA	F	G	H	T	R	**M**	S	A	F	W	D	E	S	S^P^	H	SP	S	plas	plas	plas
	NtPIP2;8t	NPA	NPA	F	G	H	T	R	M	S	A	F	W	D	E	S	S^P^	H	SP	S	plas	plas	plas
	NtPIP2;9s	NPA	NPA	F	G	H	T	R	M	S	A	F	W	D	E	S^P^	S^P^	H	S^P^	S	plas	plas	plas
	NtPIP2;9t	NPA	NPA	F	G	H	T	R	M	S	A	F	W	D	E	S^P^	S^P^	H	S^P^	S	plas	plas	plas
	NtPIP2;11s	NPA	NPA	F	G	H	T	R	M	S	A	F	W	D	E	S^P^	**N**	H	S^P^	S	plas	plas	plas
	NtPIP2;11t	NPA	NPA	F	G	H	T	R	M	S	A	F	W	D	E	S^P^	N	H	S^P^	S	plas	plas	plas
	NtPIP2;13s	NPA	NPA	F	G	H	T	R	**Q**	S	A	F	W	D	E	S^P^	N	H	S^P^	S^P^	plas	plas	plas
	NtPIP2;13t	NPA	NPA	F	G	H	T	R	Q	S	A	F	W	D	E	S^P^	N	H	S^P^	S^P^	plas	plas	plas
**NIP**	NtNIP1;1s	**NPS**	**NPA**	**W**	**A**	**V**	**A**	**R**	**F**	**S**	**A**	**Y**	**L**	**-**	**-**	**P**	**-**	**-**	**S**^**P**^	**-**	plas	plas	cyto
	NtNIP1;2s	**NPA**	NPA	W	**L**	V	A	R	F	S	A	Y	**M**	-	-	P	-	-	S	-	plas	plas	plas
	NtNIP1;2t	NPA	NPA	W	L	V	A	R	F	S	A	Y	M	-	-	P	-	-	S	-	plas	plas	plas
	NtNIP2;1s	NPA	NPA	**G**	**T**	**S**	**G**	R	**L**	**T**	A	Y	**I**	-	-	P	-	-	S^P^	-	plas	plas	plas
	NtNIP3;1s	NPA	NPA	**W**	**V**	**I**	**A**	R	**F**	**S**	A	Y	I	-	-	P	-	-	S	-	plas	plas	plas
	NtNIP4;1s	NPA	NPA	W	**F**	**V**	A	R	F	S	A	Y	I	-	-	P	-	-	S	-	plas	plas	plas
	NtNIP4;1t	NPA	NPA	W	F	V	A	R	F	S	A	Y	I	-	-	P	-	-	S	-	plas	plas	plas
	NtNIP4;2s	NPA	NPA	W	F	V	A	R	**L**	S	A	Y	I	-	-	P	-	-	**T**	-	plas	plas	plas
	NtNIP4;2t	NPA	NPA	W	F	V	A	R	L	S	A	Y	I	-	-	P	-	-	T	-	plas	plas	plas
	NtNIP4;3s	NPA	NPA	W	F	V	A	R	L	S	A	Y	I	-	-	**S**^**P**^	-	-	**S**	-	plas	tono	plas
	NtNIP5;1s	NPA	NPA	**S**	**V**	I	A	R	**F**	**T**	A	Y	I	-	-	P	-	-	S	-	plas	tono	cyto
	NtNIP5;1t	**NPS**	**NPV**	**A**	V	I	A	R	F	T	A	Y	**L**	-	-	P	-	-	S	-	plas	tono	cyto
	NtNIP6;1s	**NPA**	NPV	**S**	V	I	A	R	**L**	T	A	Y	L	-	-	P	-	-	S	-	plas	plas	plas
	NtNIP6;1t	NPA	NPV	**T**	V	I	A	R	L	T	A	Y	L	-	-	P	-	-	S	-	plas	plas	plas
	NtNIP7;1s	NPA	**NPA**	**A**	**I**	**V**	**G**	R	**Y**	**S**	A	Y	**V**	-	-	P	-	-	**T**	-	plas	plas	plas
	NtNIP7;1t	NPA	NPA	A	I	V	G	R	Y	S	A	Y	V	-	-	P	-	-	T	-	plas	plas	plas
**TIP**	NtTIP1;1s	**NPA**	**NPA**	**H**	**F**	**I**	**A**	**V**	**A**	**S**	**A**	**Y**	**W**	**-**	**-**	**T**	**-**	**-**	**-**	**-**	tono	plas	plas
	NtTIP1;1t	NPA	NPA	H	F	I	A	V	A	S	A	Y	W	-	-	T	-	-	-	-	tono	plas	plas
	NtTIP1;2s	NPA	NPA	H	F	I	A	V	A	S	A	Y	W	**-**	**-**	T	**-**	**-**	**-**	**-**	tono	plas	pero
	NtTIP1;2t	NPA	NPA	H	F	I	A	V	A	S	A	Y	W	-	-	T	-	-	-	-	tono	plas	pero
	NtTIP1;3s	NPA	NPA	H	F	I	A	V	**T**	S	A	Y	W	-	-	T	-	-	-	-	tono	plas	plas
	NtTIP1;3t	NPA	NPA	H	F	I	A	V	T	S	A	Y	W	-	-	T	-	-	-	-	tono	plas	plas
	NtTIP1;4t	NPA	NPA	H	F	I	A	V	**A**	S	A	Y	W	-	-	T	-	-	-	-	tono	plas	cyto
	NtTIP2;1s	**NPD**	NPA	H	**H**	I	**G**	**R**	**V**	S	A	Y	W	**-**	**-**	T	-	-	-	-	tono	tono	plas
	NtTIP2;1t	**NPA**	NPA	H	H	I	G	R	V	S	A	Y	W	-	-	T	-	-	-	-	tono	plas	plas
	NtTIP2;2s	NPA	NPA	H	H	I	G	R	V	S	A	Y	W	-	-	T	-	-	-	-	tono	tono	plas
	NtTIP2;3s	NPA	NPA	H	H	I	G	R	V	S	A	Y	W	-	-	T	-	-	-	-	tono	tono	Extra
	NtTIP2;3t	NPA	NPA	H	H	I	G	R	V	S	A	Y	W	-	-	T	-	-	-	-	tono	tono	plas
	NtTIP2;4s	NPA	NPA	H	H	I	G	R	V	S	A	Y	W	-	-	T	-	-	-	-	tono	plas	plas
	NtTIP2;5s	NPA	NPA	H	H	I	G	R	V	S	A	Y	W	-	-	T	-	-	-	-	tono	plas	plas
	NtTIP2;5t	NPA	NPA	H	H	I	G	R	V	S	A	Y	W	-	-	T	-	-	-	-	tono	plas	plas
	NtTIP3;1s	NPA	NPA	H	**F**	I	**A**	R	**A**	**A**	A	Y	W	**-**	**-**	**S**^**P**^	-	-	-	-	tono	plas	plas
	NtTIP3;1t	NPA	NPA	H	F	I	A	R	A	A	A	Y	W	**-**	**-**	S^P^	-	-	-	-	tono	plas	plas
	NtTIP3;2t	NPA	NPA	H	F	**V**	**G**	R	A	A	A	Y	W	**-**	**-**	S^P^	-	-	-	-	tono	plas	plas
	NtTIP4;1s	NPA	NPA	H	**H**	**I**	**A**	R	**V**	**S**	A	Y	W	**-**	**-**	**T**	-	-	-	-	tono	tono	plas
	NtTIP4;1t	NPA	NPA	H	H	I	A	R	**L**	S	A	Y	W	**-**	**-**	T	-	-	-	-	tono	tono	plas
	NtTIP5;1s	NPA	NPA	**N**	H	**V**	**G**	**Y**	**T**	S	A	Y	W	-	-	**S**	-	-	-	-	tono	plas	plas
	NtTIP5;1t	NPA	NPA	N	H	V	G	Y	T	S	A	Y	W	**-**	**-**	S	-	-	-	-	plas, vac	plas	plas
**SIP**	NtSIP1;1t	**NPT**	**NPA**	**T**	**E**	**V**	**P**	**N**	**M**	**A**	**A**	**Y**	**W**	**-**	**-**	**H**	**-**	**-**	**-**	**-**	plas	plas	Extra
	NtSIP1;2s	**NPA**	NPA	T	**G**	V	P	N	**I**	A	A	Y	W	-	-	**T**	-	-	-	-	plas	tono	Extra
	NtSIP1;2t	NPA	NPA	T	G	V	P	N	I	A	A	Y	W	-	-	T	-	-	-	-	plas	tono	ER
	NtSIP2;1s	**NPL**	NPA	**H**	**R**	**H**	**G**	**S**	**F**	**V**	A	Y	W	-	-	**S**	-	-	-	-	plas	chlo	Extra
	NtSIP2;1t	NPL	NPA	H	R	H	G	S	F	V	A	Y	W	-	-	S	-	-	-	-	plas	chlo	Extra
**XIP**	NtXIP1;6t	**NPV**	**NPA**	**A**	**-**	**T**	**A**	**R**	**V**	**C**	**A**	**F**	**W**	**-**	**-**	**S**	**-**	**-**	**-**	**-**	plas	plas	plas
	NtXIP1;6s	NPV	NPA	A	-	T	A	R	V	C	A	F	W	-	-	S	-	-	-	-	plas	plas	plas
	NtXIP1;7s	NPV	NPA	**I**	-	T	A	R	V	C	A	F	W	-	-	S	-	-	-	-	plas	plas	pero
	NtXIP1;7t	NPV	NPA	I	-	T	A	R	V	C	A	F	W	-	-	S	-	-	-	-	plas	plas	pero

Amino acid composition of NtAQPs at known functionally important positions. Listed are the two NPA motifs (NPA I in Loop B, NPA II in Loop E), ar/R residues (in Helix 2, 5, Loop C and Loop E) and the Froger’s residues (Positions 1-5). Other known regulatory residues involved with post-translational regulation of AQPs are listed: Asp28, Glu31, Ser115, His131, His193, Ser274, Ser277. Amino acid positions of these sites are relative to Spinach PIP2;1 or Arabidposis TIP2;1 (LC - His131 residue), as their crystal structures have been defined and well-studied. Serine (S) residues with a superscript P (i.e. SP) are predicted to be phosphorylated according to NetPhos3.1. Also included are the predicted subcellular localisations from Plant-mPloc, WolfPsort and YLoc; outputs include plasma membrane (plas), cytosol (cyto), tonoplast (tono), chloroplast (chlo), endoplasmic reticulum (ER), peroxisome (pero) and extracellular (extra) localisation

#### NtPIP subfamily

The NtPIPs represent the largest NtAQP subfamily with 29 members that are phylogenetically divided into PIP1 and PIP2 subgroups. Despite being the largest subfamily, the NtPIPs were among the most conserved in protein sequence (> 50%; Fig. 3b). The apoplastic exposed Loops A and Loop C were the exceptions having only ~ 20% sequence identity and varying in size between PIP1 and PIP2 proteins (Fig. 3). This sequence diversification could be of functional importance given Loop A is involved in PIP-PIP dimerization mediated primarily through a conserved cysteine residue, which is present in all NtPIPs [48, 49]. The generally high sequence similarity across most of the PIP protein domains was also reflected in both PIP1s and PIP2s having identical configuration of residues across the NPA and ar/R motifs; which were predominantly hydrophilic residues (Table 2). Only Froger’s position 2 showed variation with amino acids of different properties (G, M or Q) occupying this position (Table 2). The NtPIP1s are predominantly distinguished from NtPIP2s by having longer N-terminal and shorter C-terminal tail sequences. The N-terminal tail is involved in calcium-dependent gating of the pore which occurs through interactions involving two acidic residues (Asp28 and Glu31, Table 2) [45]. Pore gating is also triggered by pH involving protonation of a Loop D histidine (His-193, Table 2) and phosphorylation of a Loop B serine (Ser115, Table 2) [45, 47]. These four residues were identified in each NtPIPs indicating the entire subfamily retains these modes of regulation (Table 2). The Loop B serine (Ser115), or phosphorylatable threonine, was also conserved in members of XIPs, TIPs and SIPs (but not NIPs), suggesting a shared mechanism of gating regulation between different NtAQPs (Table 2). Two commonly phosphorylated serine sites were found conserved in the longer C-terminal tail of NtPIP2s (Ser274 and Ser277; Table 2, Additional file 2: Figure S5). The phosphorylation status of these serine residues are known to facilitate protein-protein interactions, influence trafficking to and from the plasma membrane, and alter the transport capacity of the pore [5, 50]. NtPIP1 proteins have the second of these serine residues (Ser277), but are not predicted to be phosphorylated (Table 2; Additional file 2: Figure S5). A strongly conserved positively charged lysine or arginine directly preceding the second phosphorylated serine is found across all NtPIPs, and also more broadly across PIPs from other plant species (data not shown), with the exception of NtPIP1;5 and PIP2;11 which have a histidine (Additional file 2: Figure S5). Histidine can achieve a positive charge through protonation, indicating a possible pH regulated functional state of the C-terminal tail in these NtPIPs.

#### NtNIP subfamily

NIPs were found to have the lowest overall sequence identity sites (~ 10%), suggesting a highly divergent subfamily at the sequence level (Fig. 3b). The sequence variation was evenly distributed across all AQP domains, with only Loop B and Loop E retaining modest conservation with > 30% identical residues per site. This comparatively higher conservation likely reflects these two loops being directly involved in forming the main pore structure and controlling substrate selectivity. Loops B and E each contain a NPA motif, and Loop E also contains ar/R and Froger’s residues (Table 2). Across the NtNIPs, there was substantial variation in the residues constituting the dual NPA motifs (NPA/S/V) and across all 5 Froger’s positions (Table 2). And all but LE2 of the ar/R residues were variable, although the residue that were present tended to be more hydrophobic (Table 2). Also notable in the NtNIPs, were their distinctively longer N and C terminals (~ 57-30aa) compared to those in other subfamilies (Fig. 3a). The extended C-terminal tail contains numerous serine residues, many of which were predicted to be phosphorylated (Additional file 2: Figure S5). Included were serine residues at homologous positions to the confirmed phosphorylated sites of Ser262 in GmNOD26 (a soybean NIP) and Ser277 in PIPs (Table 2). The Ser115 phosphorylation site that controls aspects of pore gating in PIPs was conserved and predicted to be phosphorylated in only NtNIP4;3 s, with all other NtNIPs having a structurally rigid proline residue at this position (Table 2).

#### NtTIP subfamily

Conservation among the NtTIPs was ~ 22% sequence identity (Fig. 3b). Similar to the NIPs, the highest sequence conservation occurred in Loops B and E (> 40%). The dual NPA motif, ar/R H2 and Froger’s P3 to P5 are well conserved among the different TIP subgroups. The exceptions being NtTIP2;1 s with a NPD configuration of the first NPA motif, and the NtTIP5;1 proteins which have a H > N substitution at ar/R H2 (Table 2). The other ar/R and Froger’s sites are rather variable among the NtTIPs, especially ar/R LE2 which varies between amino acids of quite differing properties (V, R or Y; Table 2). A histidine opposed to phenylalanine located at ar/R LC of NtTIP2s, TIP4s and TIP5s (Table 2), suggests an enhanced capacity to transport ammonia [51]. The Ser115 phosphorylation site that controls pore gating in PIPs was identified in 5 of the 22 NtTIPs, with the remaining NtTIPs possessing a threonine which is also a potentially phosphorylatable residue. NtTIP2 and NtTIP5 proteins have a conserved histidine (His131) in Loop C that is involved in a similar pH regulated gating of the pore to that of His193 in Loop D of PIPs and NIPs [52, 53]. The C-terminal tail of NtTIPs contained on average less than 2 serine residues, none of which were predicted to be phosphorylation targets (data not shown).

#### NtSIP subfamily

While only comprising of 5 genes, the NtSIP subfamily had low sequence conservation, with Loop A the least conserved (Fig. 3b). The first NPA motif varied with NPA/T/L combinations (Table 2). Substantial variation was also was found in other key residues with completely different configuration of residues in the ar/R and Froger’s P1-P2 between NtSIP1 and NtSIP2 proteins (Table 2). The N- terminal tail of NtSIPs were distinctly shorter than other subfamilies (~7aa) (Fig. 3a).

#### NtXIP subfamily

The XIPs are a small sub-family with high sequence identity (~ 75%). The first NPA motif is replaced by a NPV motif in all four NtXIP proteins (Table 2). There is a strong consensus in the residues residing in the Froger’s and dual NPA motifs, with the only variation being I/A at ar/R H2 (Table 2). Concordant with other studies of XIPs, the loop C of NtXIP is substantially longer (~38aa) compared to that of other subfamilies [54]. NtXIPs have the conserved phosphorylated Ser115, although it was not a predicted phosphorylation target (Table 2). The C-terminal tail of NtXIPs contained a single serine residue which was not predicted to be phosphorylated (data not shown).

### Subcellular localisation of tobacco AQPs *in planta*

AQPs can facilitate diffusion of a range of substrates across various plant membranes and the specific membrane localisation can vary between the different subfamilies, which ultimately influences sub-cellular flow and compartmentalisation of solutes. Computational prediction programs can be used as an initial inference of subcellular localisation to further help elucidate putative biological activities and physiological functions of candidate proteins [55]. We conducted subcellular prediction analyses using three commonly used software programs, Plant-mPLoc, Wolf Psort and YLoc (see materials and methods). Consistency in prediction across the three programs was found for 35 (46%) of NtAQPs (Table 2). Consensus in predicted localisation was mainly observed for the PIP2s and the NIPs, which were generally predicted to be plasma membrane (PM) localised. The TIPs and SIPs appeared to have the most contrasting predictions in subcellular localisation results, with TIP localisations ranging between tonoplast, PM, peroxisome, cytoplasmic and extra cellular localisation; and SIPs having PM, tonoplast, chloroplast, ER and extra cellular localisations across the 3 prediction tools (Table 2).

**Fig. 4:**
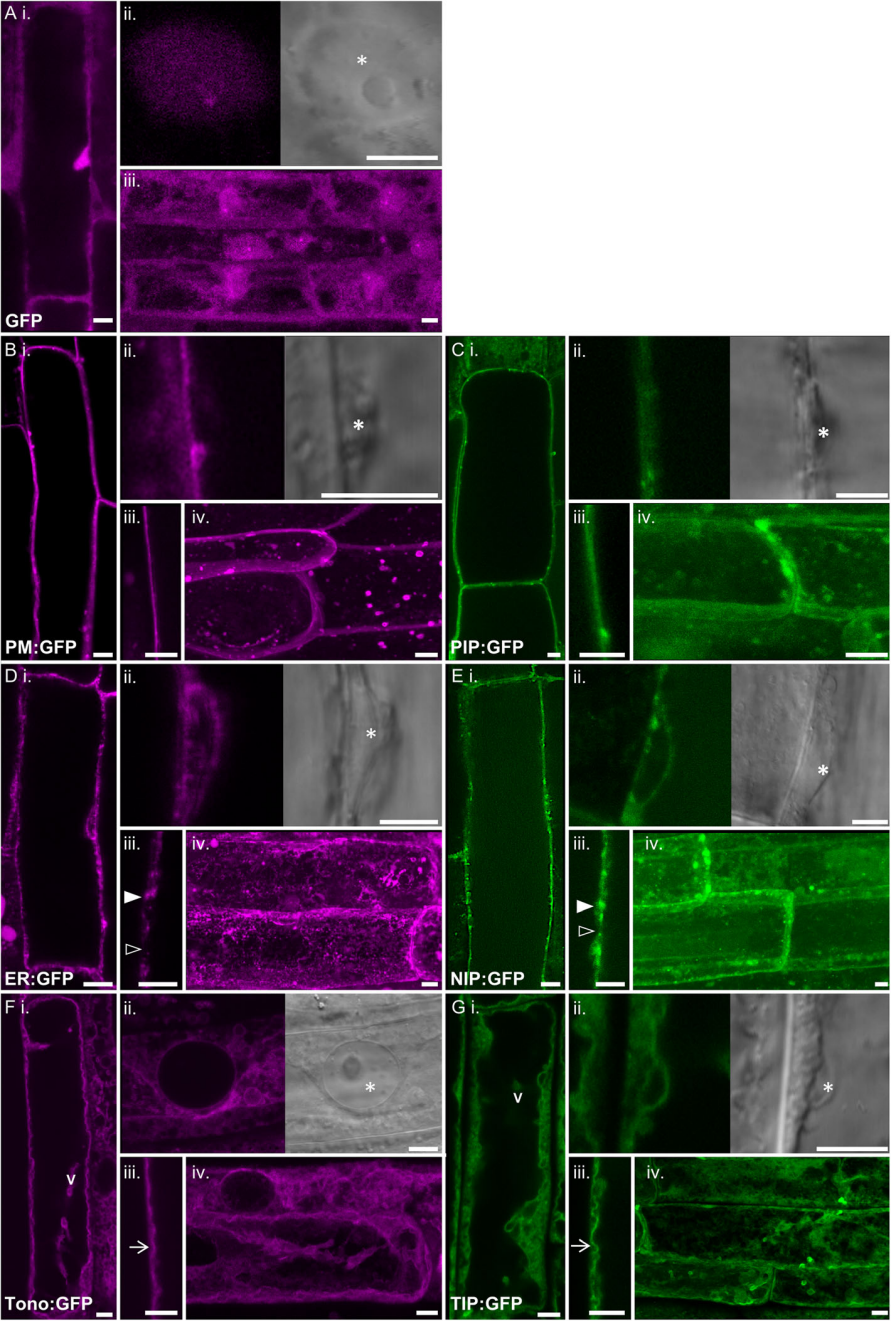
*In planta* sub-cellular localisation of PIP, TIP and NIP aquaporins. Confocal images of root cortical cells of transgenic 8-day-old Arabidopsis seedlings. **a, b, d, f** GFP marker lines; false coloured purple. **c, e, g** NtAQP:GFP lines; false coloured green. Subpanels (i-iv) are; (i.) Optical cross-section midway through a root cortical cell. (ii) GFP signal associated with nucleus; confocal image (left) DIC image (right). (iii.) Close-up of cell peripheral margin. (iv.) Maximum intensity projections compiled from serial z-stack images. **a** GFP-only localisation. **b** Plasma membrane (PM:GFP) marker. **c***NtPIP2;5 t* (PIP:GFP). **d** Endoplasmic reticulum (ER:GFP) marker. The ER is known not uniformly be present around the cell periphery which is reflected by regions of bright GFP signal (solid arrowhead) interspersed with regions of no GFP signal (open arrowhead). **e***NtNIP2;1 s* (NIP:GFP). **f** Tonoplast (Tono:GFP) marker showing characteristic features of the tonoplast membrane including, transvacuolar strands (v) and general undulating appearance (arrow). **g***NtTIP1;1 s* (TIP:GFP). Notable sub-cellular features are marked by a; asterisks for the nucleus, ‘V’ for transvacuolar strands, arrowheads indicate instances of varied brightness (solid = high signal, empty = no signal) in GFP fluorescence in **d** (iii) and **e** (iii), or undulations of the tonoplast in **f** (iv) and **g** (iv). Scale bar 5 μm

To complement the predictions, representative tobacco AQPs from the larger PIP, TIP and NIP subfamilies were visualised *in planta* using GFP:NtAQP fusions. NtSIPs were not included in this analysis as they are a smaller AQP subfamily, while NtXIPs are already established as localising to the PM [56]. Plant AQPs retain their capacity for faithful subcellular localisations between tissues, even when translocated across plant species, as evident from numerous studies examining subcellular localisation or physiological manipulation using transgenic AQPs foreign to the host species [5, 57–62]. As such, we introduced our tobacco GFP:AQP transgenes into Arabidopsis, to be able to utilise established GFP marker lines that delineate specific subcellular compartments [63]. Such marker lines are crucial in guiding the correct interpretations of subcellular locations, given the close proximity of certain subcellular structures occupied by AQPs. For example, both the PM and ER are possible locations, but parts of the ER network lay immediately adjacent to the PM, making it difficult to discern between ER, PM, or co-localisation. Interpretations are further compounded by the large vacuoles of most plant cells that occupy much of the internal volume, pushing the cytoplasm and its contents to the periphery. This can give the illusion of PM localisation even for cytosolic proteins such as ‘free’ GFP, especially if only examined as a 2D-optical slice at the whole cell level (Fig. 4ai).

We used confocal microscopy to visualise the subcellular localisation of GFP:NtAQP and GFP marker lines using both 2-D slices and 3-D optical stacks. To avoid signal contamination from chlorophyll auto-fluorescence, which excites and emits at wavelengths close to GFP, we examined root cells. GFP marker lines localising to the cytoplasm, plasma membrane (PM), ER, and tonoplast (tono), were used as these are the expected possible locations of the PIPs, TIPs, or NIPs (Fig. 4). Key differences between the four sub-cellular features were clearly discernible in the vicinity of the nucleus, the topography of the signal, and 3D renders of serial Z-stack images of the cells (Fig. 4b-g). The PM:GFP marker localised exclusively to the periphery of the cell when adjacent to the nucleus (Fig. 4bii), the ER:GFP wrapped around the nucleus (Fig. 4dii), and Tono:GFP localised internally to the nucleus leaving a signal void on the side adjacent to the PM (Fig. 4fii). PM:GFP produced a sharp defined integration with the cell margin (Fig. 4biii), featuring as an outer shell in the 3D render (Fig. 4biv). The ER:GFP peripheral signal was mottled in appearance (Fig. 4di), consisting of localised bright specks with distinct regions of no signal (Fig. 4diii), that appeared as a ‘web’ in the 3D render (Fig. 4div). Tono:GFP was present as large undulating ‘sheets’ of signal associated with the trans-vacuolar strands (tonoplast-delimited cytoplasmic tunnels) and folds of vacuole membrane (tonoplast) (Fig. 4fi-iv), which had a distinct ‘wavy’ topography (Fig. 4fiii).

Having established the defining features of the marker lines, we moved to examining the representative NtAQPs. Distinct *in planta* subcellular localisation patterns were observed for the PIP, TIP and NIP NtAQPs, consistent with the known membrane targeting properties of these different AQP subfamilies (Fig. 4c,e,g). The GFP signal of the representative PIP (NtPIP2;5 t) appeared sharp and uniformed around the cell periphery, with signal running external to the nucleus and forming a smooth outer shell in the 3D render with no discernible signal in any internal structures (Fig. 4c). This pattern was concordant with a PM:GFP marker (Fig. 4b), indicating a strong integration of NtPIP2;5 t into the PM.

The representative NtNIP (NtNIP2;1 s), had features indicating it co-localises to the PM and ER. The peripheral localised NtNIP GFP signal was mottled in appearance with distinct specks of intense bright signal similar to the ER marker. However, unlike the ER marker, these specks were dispersed along a consistent basal signal continuous around the cell periphery, indicative of PM localisation (Fig. 4ei-iii). The 3D render further demonstrated the shared shell-like PM signal overlapping the mottled web-like ER patterned signal (Fig. 4eiv).

The localisation of the representative NtTIP (NtTIP1;1 s) is consistent with integration into the tonoplast. The NtTIP GFP signal showed a uniform yet diffuse localisation within the cell consistent with tonoplast labelling. The NtTIP GFP signal surrounded the nucleus on the cytosolic but not plasma membrane side (Fig. 4gi-ii), and the labelled membrane had a wavy topography with the occurrence of internal membranes resembling transvacuolar strands (Fig. 4giii-iv).

The PM integration of *NtPIP2;5* was predicted by all 3 software programs, whereas the tonoplast localisation of *NtTIP1;1 s* was only predicted by Plant-mPLoc. Lastly, the PM localisation of *NtNIP2;5 s* was anticipated in all 3 programs, but none predicted its co-localisation with the ER (Table 2).

### Parental association and recent evolutionary history of tobacco AQPs

**Fig. 5:**
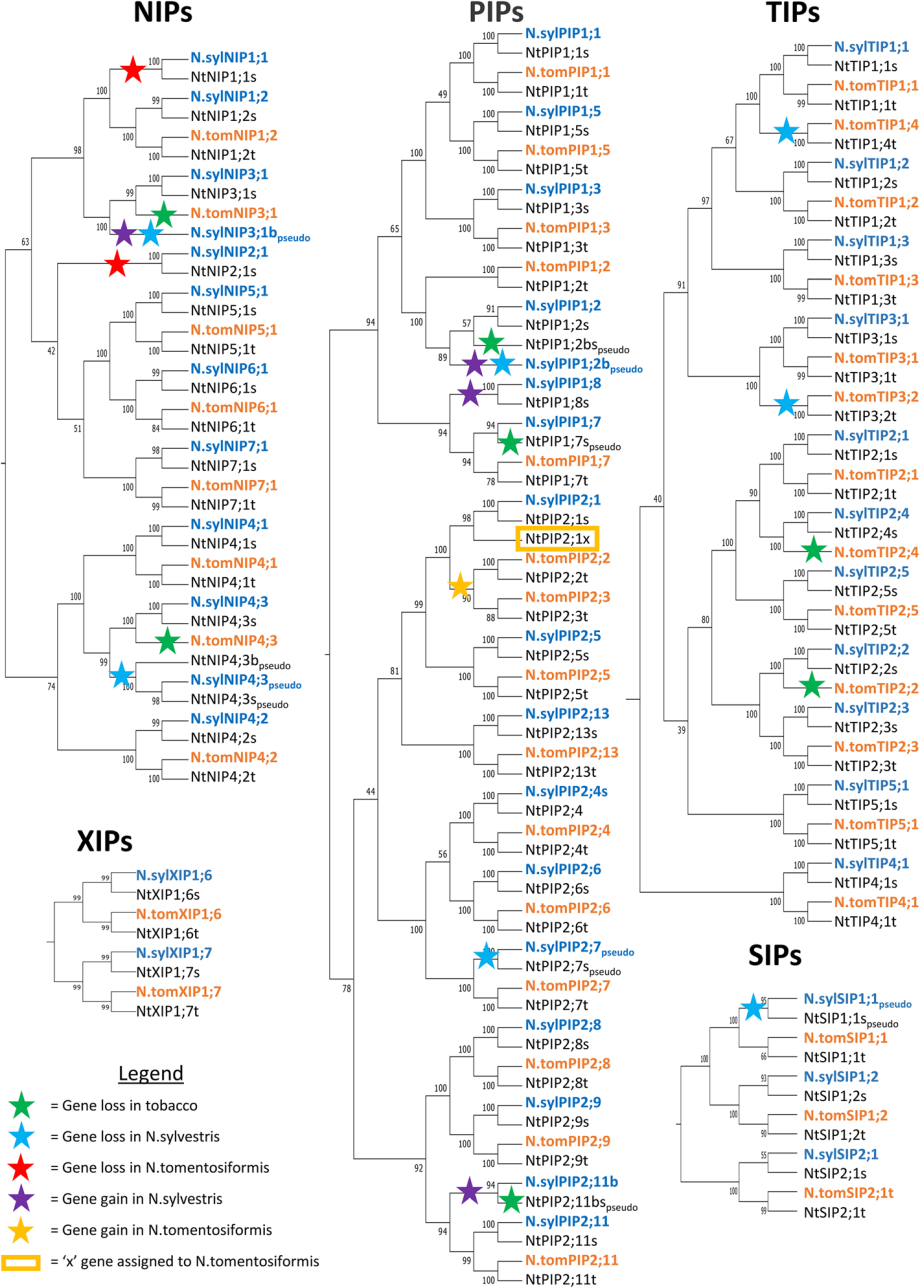
Phylogenetic relationship of tobacco, *N. sylvestris* and *N. tomentosiformis* AQPs. Phylogenetic trees for each AQP sub-family were generated using the neighbour-joining method from MUSCLE alignments of nucleotide coding sequences. Confidence levels (%) of branch points generated through bootstrapping analysis (*n* = 1000). *N. sylvestris* (N. syl) and *N. tomentosiformis* (N. tom) AQPs are colour coded in blue and orange, respectively. Green stars indicate a loss of a parental gene in tobacco post-hybridisation; Blue and Red stars indicate gene loss events in *N. sylvestris* and *N. tomentosiformis*, respectively. Purple and Yellow stars indicate pre-hybridisation gene gain events in *N. sylvestris* and *N .tomentosiformis,* respectively

The distinctive phylogenetic pairing of most NtAQPs in our initial phylogenetic characterisation, is likely characteristic of the recent evolutionary origin of tobacco, which arose from an allotetraploid hybridisation event between *N. sylvestris* and *N. tomentosiformis* only ~ 0.2 M years ago [42, 43]. To explore the evolution of the tobacco AQP family, we identified the AQP gene families in the two parental lines using NtAQP nucleotide coding sequences as queries in BLAST searches. Initially, 40 and 41 AQPs were identified in both *N. sylvestris* and *N. tomentosiformis* respectively, which is comparable to the number of AQP genes found in the related diploid species of tomato and potato (Table 3). As shown in this work, tobacco has 76 AQPs, almost a full set from each parental species (40 *N.sylvestris*, and 42 *N.tomentosiformis*), being consistent with a recent allotetraploid hybridisation event. The introduction of the parental *N. sylvestris* and *N. tomentosiformis* AQPs into the NtAQP phylogeny, transformed the majority of the distinct NtAQP phylogenetic pairs into small clades of four genes where each of the paired NtAQPs was now clearly associated with an AQP from one of the two parents (e.g. NtPIP1;1 sub-clade, Fig. 5). This phylogenetic relationship confirmed that the distinctive phylogenetic pairing of NtAQPs corresponds to orthologous ‘sister’ genes arising from hybridisation, with both parental genomes having contributed one AQP gene to each tobacco sister pair (Fig. 5). Initially 30 sister gene pairs were identified that had a clear match to an orthologous gene from both *N. sylvestris* and *N. tomentosiformis* (Fig. 5). The ancestral origin of the *NtAQP* genes were denoted in the nomenclature by the addition of a suffix ‘s’ or ‘t’ (e.g. *NtPIP1;1 s* and *NtPIP1;1 t*), to indicate a *N. sylvestris* or *N. tomentosiformis* lineage, respectively.

**Table 3:** Summary of total AQPs currently identified within Solanaceae

Solanaceae species	PIPs	TIPs	NIPs	SIPs	XIPs	Total AQPs	Reference
***S.lycopersicum*(tomato)**	14	11	10	4	6	45	Reuscher et al. 2013 [40]
***S.tuberosum*(potato)**	15	11	10	3	4	47	Venkatesh et al. 2013 [41]
***N.sylvestris***	15	11	10	2	2	40	This study
***N.tomentosiformis***	16^(15)^	13	8	3	2	42^(41)^	This study
***N.tabacum*(tobacco)**	29	22	16	5	4	76	This study

**Table 3** Summary of total AQPs currently identified within Solanaceae. Tomato and potato AQP families were characterised by Reuscher et al. (2013) and Venkatesh et al. (2013), respectively. *N.sylvestris*, *N.tomentosiformis* and tobacco gene families were established in this study. Numbers indicate genes occurring in each AQP sub-family (PIP, TIP, NIP, SIP and XIP). The number of identified N.tomentosiformis PIPs through the BLAST searches was 15 (noted by superscript value), however, we predict the total number of PIPs at the time of hybridisation to be 16 due to NtPIP2;1x likely being of *N.tomentosiformis* origin. This brings the overall number of N.tomentosiformis AQPs to 42

One *NtAQP* gene had no resolved match to a *N. sylvestris* or *N. tomentosiformis* parental AQP and was assigned a suffix ‘x’ (*NtPIP2;1x*). The lack of a clear parental match to *NtPIP2;1x* likely means that the orthologous gene has been lost in the parental genome post tobacco emergence, or the orthologous parental AQP was not identified due to incomplete coverage of sequencing data. Either way, the presence of this gene in the tobacco genome allows us to infer its presence in a parental genome at the time of hybridisation. We predict that *NtPIP2;1x* was inherited from *N. tomentosiformis*, as it occurs in a distinct clade with a tobacco sister gene (*NtPIP2;1 s*) and an orthologous *N. sylvestris* AQP (*N.sylPIP2;1*), but lacks a *N. tomentosiformis* progenitor ortholog (orange box, Fig. 5). As such, assigning *NtPIP2;1x* as a *N. tomentosiformis* descendant, brings the total number of AQPs in the parental genomes to 40 in *N. sylvestris* and 42 in *N. tomentosiformis*, with the total number of genes within the PIP, NIP and TIP subfamilies being very similar to those of tomato and potato (Table 3).

The phylogenetic analysis also revealed recent evolutionary events in the tobacco*, N. sylvestris* and *N. tomentosiformis* AQP families. These events were recognised by deviations from the conventional four-gene small sub-clade groupings comprised of the tobacco sister genes and their respective parental orthologs. Seven *AQP* gene loss events were recognised in *N. sylvestris*, six of which occurred prior to the tobacco hybridisation event as the given *AQP* was absent in both *N. sylvestris* and tobacco (blue stars, Fig. 5). In several cases, the remnants of the eroding *N. sylvestris* pseudo gene were also inherited and identifiable in the tobacco genome (e.g. *SIP1;1* and *PIP2;7*; Fig. 5). Two gene loss event was recognised in *N. tomentosiformis*, with no representative *NIP1;1* or *NIP2;1* orthologs identified in either *N. tomentosiformis* or tobacco (red star, Fig. 5). Five parental AQP genes have been lost in tobacco, three from *N. tomentosiformis* and two from *N. sylvestris* origins (green stars, Fig. 5). Instances of gene gains were also evident in both parental species prior to the tobacco hybridisation event (purple and orange stars, Fig. 5). These gained genes were distinct in the phylogenies as they did not uniquely match a specific Solanaceae gene ortholog, appearing instead as a duplicate copy of an existing *AQP* gene within the tobacco parental species (Additional file 2; Figure S4). Four AQP gene gain events occurred in *N. sylvestris*, two of which (*N.sylNIP3;1* and *N.sylPIP1;2*), began redundant gene erosion prior to tobacco hybridization (purple stars, Fig. 5). The third, *N.sylPIP2;11b*, is retained as a functional unit in *N. sylvestris* but has eroded in tobacco; hence the designation ‘b’ as opposed to a unique numerical identifier. The fourth gene, *N.sylPIP1;8*, has been retained in both *N. sylvestris* and tobacco as a functional gene (purple star, Fig. 5). A single gene duplication event was recognized in *N. tomentosiformis*, giving rise to *PIP2;2* and *PIP2;3* orthologs which were both inherited and subsequently retained as functional genes in tobacco (orange star, Fig. 5).

### Tobacco AQP gene expression

#### The NtAQP transcriptome dataset

To provide insight into possible physiological roles of the various NtAQP isoforms, publicly available whole transcriptome RNA-seq datasets [42, 43] were processed and analysed to compare organ-specific expression patterns of the 76 tobacco AQPs. Although, all datasets had great read depth (100–200 million paired reads per tissue), the Sierro et al. (2014) transcriptome of the TN90 cultivar was chosen for analysis, as it provided the most extensive sampling of different tissues at various developmental stages (young leaf, mature leaf, senescent leaf, stem, root, young flower, mature flower, senescent flower and dry capsules).

Although the *NtAQP* sister genes are highly homologous in their nucleotide coding sequences (~ 96.5%), the SNPs that are present occur at a frequency and distribution enabling unique mapping of reads to differentiate between sister genes. In the TN90 dataset, we detected expression from 75 out of 76 NtAQPs, with only *NtXIP1;4 t* having no mapped mRNA reads. However, *NtXIP1;4 t* is an expressed gene, albeit at very low levels, as indicated by the low transcript abundance detected in the K326 cultivar (data not shown). To validate the accuracy of the *NtAQP* expression profiles, we compared it to RNA-seq data from *N. sylvestris* and *N. tomentosiformis*; with the assumption that the majority of AQP orthologs will have retained similar expression profiles between these closely related species. The parental datasets are independently derived from those of the tobacco dataset, and sampled root, leaf and floral tissues at substantial read depths (~ 265 million paired reads per tissue) [64]. Correlations of relative transcript abundances was compared in two-dimensions; (i) between AQPs within a given tissue and (ii) a given AQP across tissues (Additional file 2: Figure S6). Within equivalent tissues, the relative transcript abundance of *N.sylAQP* vs. *NtAQPs* and *N.tomAQP* vs. *NtAQPt* genes, correlated well (R^2^ root, leaf, flower: 0.91, 0.74, 0.98 and 0.65, 0.74, 0.80, respectively). Across tissues, the majority (> 80%) of NtAQPs and NtAQPt genes showed matching expression profile to their respective parental orthologs (Additional file 2: Figure S6). As expected, the relative transcript abundance between *AQP* sister genes within tobacco (i.e. *NtAQPs* vs. *NtAQPt*), correlated better than orthologs between parental lines (i.e. *N.sylAQP* vs. *N.tomAQP*) (Additional file 2: Figure S6). Overall, the largely conserved patterns indicate that the tobacco transcriptome data provides a suitably accurate representation of the NtAQP transcriptome.

#### Profiling the NtAQP transcriptome

**Fig. 6:**
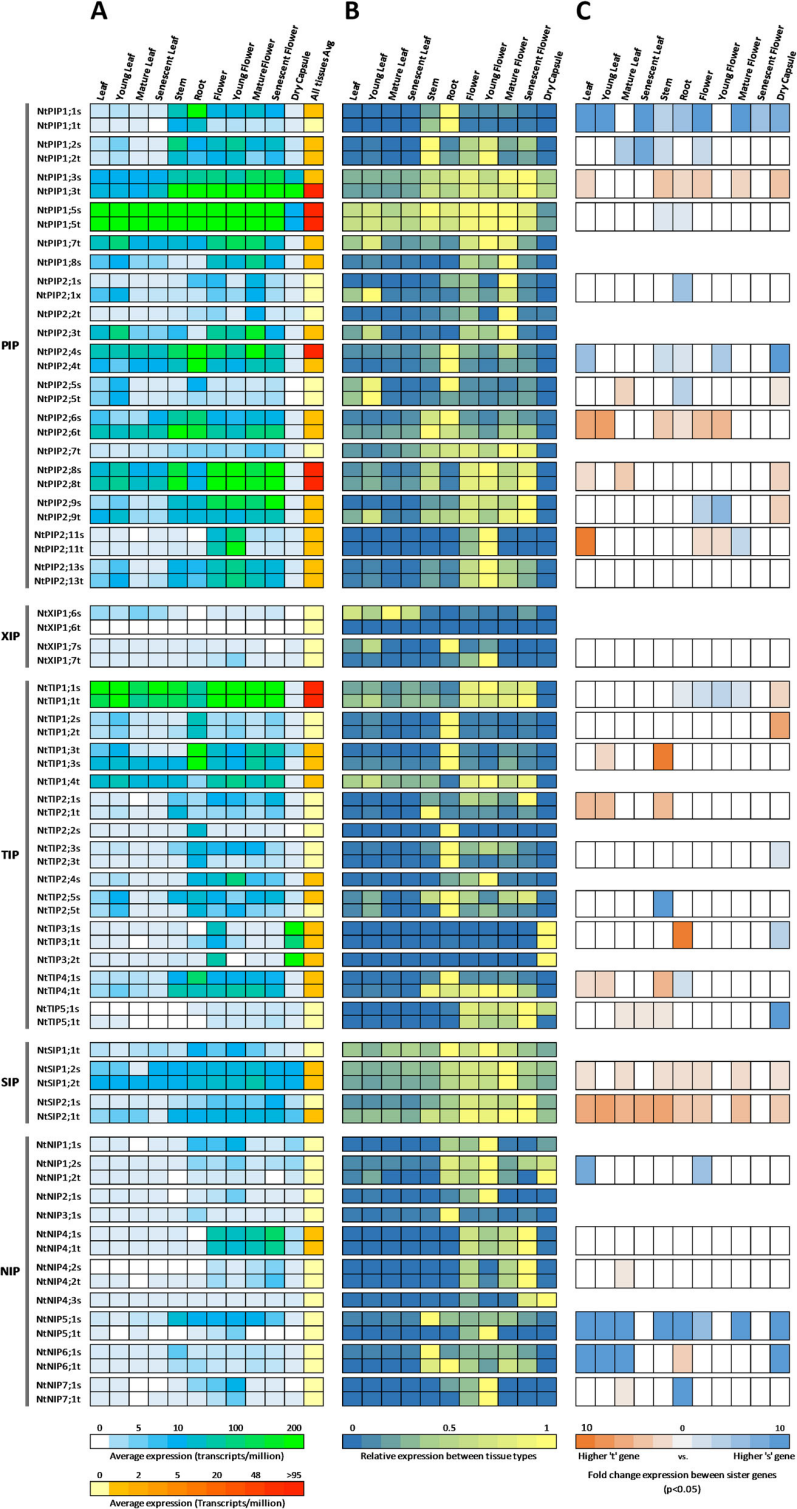
Expression patterns of NtAQP genes in different tissues. **a** Absolute NtAQP gene expression. Heatmap of gene expression (transcripts per million) of NtAQPs across different tissues. Green shading represents higher expression, graduating to a light blue for lower expression, as per key. Included in the last column is the average gene expression across all tissues examined; red shading for high expression moving towards yellow for low expression, as per key. **b** Relative expression compared to the highest expressing tissue for the given NtAQP. Heatmap of tissue-specific gene expression with values standardised to the tissue showing the highest expression for that given NtAQP. Yellow indicates high expression graduating towards blue for low expressing tissue. **c** Comparison of expression patterns between AQP sister genes. Heatmap of significant fold change differences in expression (*p* < 0.05) between sister genes across the different examined tissues. Blue indicates higher expression of the ‘s’ gene and orange higher expression of the ‘t’ gene

Among the NtAQP subfamilies, gene expression of PIPs and TIPs was generally greater than for SIPs, XIPs and NIPs (Fig. 6a). Among the most highly expressed *NtAQPs*, *PIP1;5 s*, *PIP1;5 t, PIP1;3 s* and *PIP1;3 t* stood out as being constitutively expressed in all major plant organs, while *TIP1;1 s* and *TIP1;1 t*, were present in all tissues except for the dry capsule (Fig. 6a). Some highly expressed genes also showed a level of tissue specificity, with *NIP4;1 s* and *NIP4;1 t* expressed only in flowers, and *TIP3;1 s, TIP3;1 t* and *TIP3;2 t* predominantly in the flower capsule (Fig. 6a).

To examine differential expression between plant organs, the expression levels of a given *AQP* were standardised relative to its highest expressing tissue (Fig. 6b). *AQP*s with a broad expression distribution throughout the plant could be readily identified (e.g. *SIP1;2* and *PIP1;5* sister pairs, Fig. 6b). Other AQPs show tissue specific expression: young flowers (*PIP2;11 s* & *PIP2;11 t*; *NIP2;1 s*), leaves (*PIP2;5 s* and *PIP2;*5 t; XIP1*;6 s*; *PIP2;1x*) or roots (*TIP1;*2, *TIP1;3*, *TIP2;2*, and *TIP2;3* genes). At the sub-family scale, *NtNIPs* and *NtTIPs* are found to be preferentially expressed in roots, stems and flowers, with a low tendency for expression in leaves (Fig. 6b). *NtPIPs* and *NtSIPs* are more broadly expressed, while there is no expression of *NtXIPs* in either the stem or dry capsule (Fig. 6b). Within subfamilies we see gene members with specialised or preferential tissue expression. For example, some NtPIPs preferentially expressed in the roots (*PIP1;1 s & PIP1;1 t*; *PIP2;4 s* & *PIP2;4 t*), others express preferentially in leaves (e.g. *PIP2;5 t* & *PIP2;1x*), while *PIP2;11 s* & *PIP2;11 t* have become specialised in young flowers (Fig. 6b). Discrete tissue-specific specialisation was also observed for members of the other families, for instance, *TIP3;1* and *TIP3;2* genes express only in dry capsule (seeds), and expression of *NIP4;1* and *NIP4;2* was only detected in flowers (Fig. 6b).

Next we compared differences in expression between sister genes to explore possible functional divergence. In general, sister gene pairs showed matching patterns of tissue-specific expression (Fig. 6b). However, of the 31 proposed sister gene pairs, 18 showed notable differential expression levels in at least one tissue (Fig. 6c). In the majority of these instances a single sister gene of the pair was more highly expressed in several plant organs. Examples include, *NIP5;1 s, SIP2;1 t, SIP1;2 t, PIP2;6 t, PIP2;4 s, PIP1;3 t* and *PIP1;1 s*. There were also several instances of contrasting expression where sister genes show distinctions in preferential expression between plant organs. For example, *TIP3;1 s* with 4-fold higher expression in the capsule compared to its sister pair *TIP3;1 t*, which is expressed > 10-fold higher in roots (Fig. 6c). Further examples of contrasting expression include, *NtPIP2;5 t* (leaves) against *NtPIP2;5 s* (roots) and *NtNIP6;1 s* (leaves and dry capsule) against *NtNIP6;1 t* (roots) (Fig. 6c).

#### Conservation with other Solanaceae species

As a means of exploring conservation in biological activities and physiological functions between AQP orthologous of different species, we compared tissue-specific expression levels of NtAQPs with their orthologs from the closely related tomato and potato species. This was done by comparing the relative gene expression across root, leaf and floral tissues of *AQP* genes we have identified as being orthologs between the Solanaceae species (e.g. *NtPIP1;1 s* & *NtPIP1;1 t* in tobacco, *SlPIP1;1* in tomato and *StPIP1;2* in potato; listed Additional file 1: Table S2). We were able to perform this analysis on the PIPs, TIPs, NIPs and SIPs but not the XIPs given the previously mentioned difficulty of assigning orthology between the species. Even randomised pairwise comparisons of expression patterns between NtXIPs with those of tomato and potato, could not find consensus patterns, hinting further towards the unique intra-species diversification of XIPs within the Solanaceae (Additional file 2: Figure S7).

**Fig. 7:**
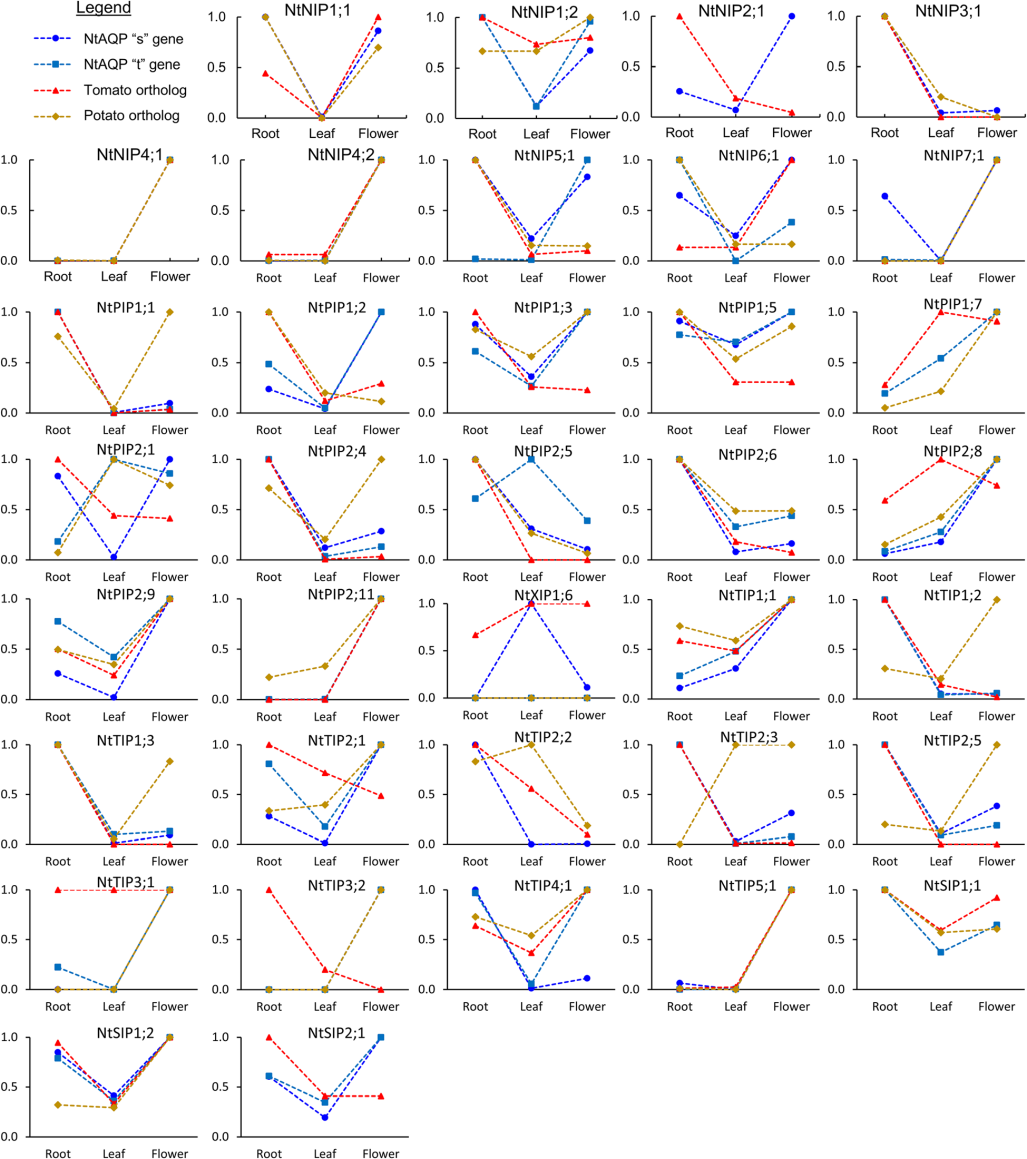
Tissue-specific gene expression patterns of AQP isoforms in tobacco, tomato and potato. Graphs contain relative gene expression (standardised to highest expressing tissue) across root, leaf and flower tissues for tobacco sister genes (light and dark blue) and their corresponding tomato (red) and potato (brown) orthologs as listed in Additional File 1: Table S2

In the majority of instances (25 of 36 Solanaceae AQP ortholog sets), the tobacco sister genes had similar patterns of relative expression levels between the three organs to their orthologs from both tomato and potato, implying conserved physiological roles for the orthologs across the Solanaceae family (e.g. *NIP1;1*, *NIP3;1*, *NIP4;*2, *PIP2;6*, *PIP2;9*, *PIP2;11*, *TIP5;1*, and *SIP1;1*; Fig. 7). Some deviations in tissue-specific expression patterns were observed between orthologs, suggesting possible species-specific functional diversification. The predominant observed deviations were instances where either the tobacco, tomato or potato AQP differed in their tissue-specific expression pattern compared to the orthologs from the other Solanaceae. Examples include; the tobacco *NtNIP5;1*, *NtPIP1;2, TIP1;1*; the tomato *SlPIP2;8*, *SlTIP2;*1, *SlTIP3;1,* and *SlTIP3;2* genes; and the potato *StPIP1;2* (*NtPIP1;1* ortholog), *StTIP1;2, StTIP1;1* (*NtTIP1;3* ortholog) and *StTIP2;4* (*NtTIP2;3* ortholog) genes (Fig. 7)*.* Additionally, we observed one case where a NtAQP sister gene (*NtPIP2;5 s*), differed in expression from the tomato, potato and its NtAQP “t” sister gene; suggesting a potential diversification in gene function within tobacco. More complex deviations were also observed involving tobacco sister genes having contrasting expression to each other, that matched a similar contrast in expression between the tomato and potato orthologs (e.g. *NtPIP2;1* and *NtNIP6;1* sister genes; Fig. 7).

## Discussion

The growing amount of research into AQPs is greatly advancing our understanding of their diversity and functional roles, towards manipulating them to potentially enhance plant performance and resilience to environmental stresses [5, 29, 31, 65, 66]. The establishment of the tobacco AQP gene family allowed us to efficiently contribute to the current knowledge of AQP biology by, comparing regions of homology within and across closely related species, analysing pore-lining residues, identifying key structural characteristics, and providing necessary information and candidates for future functional screens. Furthermore, elucidating orthology between the already characterised tomato [40] and potato [41] AQPs, enables comparisons between isoforms across these Solanaceae species, which will facilitate the translation of knowledge from tobacco into its closely related and horticulturally important crop species.

### NtAQP protein sequence analysis and associations with AQP function

We found that the tobacco AQP family comprises of 76 members, making it one of the largest AQP families characterised to date; second only to the polyploid canola (*Brassica napus*) with 121 members [14, 15]. The 76 NtAPQs include members of each of the five major AQP subfamilies common to angiosperms (i.e. NIPs, PIP, TIPs, SIPs, and XIPs). Correctly defining and analysing the NtAQP protein structures, sequence homology, and comparison of functionally relevant residues, helps towards predicting potential permeating substrates, post-translational regulation, and subcellular localisations. AQP monomers have a highly conserved structure, with transmembrane (TM) segments providing a structural scaffold and defining the channel environment, with the connecting loops also having significant roles in channel function [45]. We found a high conservation in length and sequence identity of the NtAQP TM domains; their variability likely constrained to maintain structural integrity of the AQP monomer [67]. Additionally, conservation of critical residues in TM domains is essential for tetramer formation, with modifications leading to aberrant AQP oligomerisation [68]. NtAQP loops and termini had notable differences in lengths and lower sequence conservation across subfamilies; such variation has implications for AQP monomer interactions, pore accessibility and cellular membrane destinations [54, 69].

AQP solute selectivity are conferred through specific structural features of the AQP monomer’s pore, and substrate interactions with pore-lining residues. We surveyed known specificity-determining residues across the NtAQPs, including the aromatic arginine (ar/R) filter, NPA domains, and Froger’s positions [7, 8, 11, 70]. We observed an increased sub-family conservation in the loops harbouring these specificity-determining residues, in particular Loops B and E which have a direct role in forming the transmembrane pore. Each subfamily had their unique characteristic combination of amino acids at these locations concordant with known subfamily substrate specificities. For example, NtPIPs have more polar residues in their ar/R filter which is consistent with PIPs in general having the propensity to permeate water, whereas the NtNIPs have more hydrophobic amino acids in their ar/R filter, consistent with their poorer water permeability and preference for substrates such as ammonia, urea and metalloids instead [7, 11].

Additional to the specificity-determining pore lining residues, post-translational modification of specific residues (e.g. through protonation or phosphorylation), also directly or indirectly determine the transport mechanics of the AQP monomer [71]. Plants rely on these secondary mechanisms to ensure tight regulation of AQPs, especially in response to stresses. Gating of the monomeric pore in response to external stimuli is a key control over AQP function. Among currently characterised residues involved in gating (listed in Table 2), we found subfamily-specific conservation across the NtAQPs. For example, all NtPIPs had the Loop D Histidine (His193) which is highly conserved across all plant PIPs, and can be protonated in response to changes in cytosolic pH (e.g. flooding induce hypoxia), and leading to the closure of the PIP pores [47]. pH regulated responses are important for AQP as is the C-terminal tail of the PIP proteins [71]. These facts drew our attention to the identified Lysine/Arginine > Histidine substitution in the C-terminal tails of *NtPIP1;5* and *NtPIP2;11* (Addition file 2: Figure S5). The normally positively charged Lysine/Arginine residue present in all other NtPIPs, and highly conserved across plant PIPs in general, directly precedes a functionally important phosphorylated serine. Together this suggests a likely functional relevance of a positively charged residue at this position in PIP regulation. The Histidine present at the equivalent position in NtPIP1;5 and NtPIP2;11 can still obtain the conserved positive charge upon protonation, implying a possible novel pH control over the regulatory influences normally imposed by the PIP C-terminal tail.

Some sharing of gating mechanisms between NtAQPs from different subfamilies can be inferred from our analysis. For example, the Loop B serine (Ser155) which in PIPs is involved in phosphorylation dependent disruption of N-terminal tail gating [45, 46], is conserved in some members of the other NtAQP subfamilies. NtPIPs and NtNIPs both seem to be regulated by phosphorylation in their C-terminal tails given the abundance of serine residues. The phosphorylation state of the C-terminal tail is known to regulate channel activity and also control trafficking to the plasma membrane [46, 72]. Interestingly, the NtTIPs had a dearth of serine residues in the C-terminal tail, suggesting a lack of a C-terminal phosphorylation-dependent regulation mechanism. This perhaps is due to differences in functional requirements being integrated into the vacuole membrane versus the plasma membrane integration of PIPs and NIPs. Consistent with differing regulatory requirements, we found that NtTIP2 and NtTIP5 proteins possessed a conserved histidine (His131) in loop C that is involved in a similar pH regulated gating of the pore to that of His193 in Loop D of PIPs and NIPs [52, 53] (erroneously reported as located in loop D of VvTnTIP2;1 in Leitão et al., 2012). However, unlike the cytosolic PIP/NIP Loop D His193, the TIP Loop C His131 is likely orientated into the vacuole and thus responding to the vacuole contents and environment.

Other structural features NtAQP of note include: the longer Loop D of PIPs compared to the other subfamilies which aids in its ability to cap the pore entrance [45]; the substantially longer Loop A of PIPs compared to the other NtAQPs, known to play a role in tetramer formation by mediating disulphide bonds between PIP1 and PIP2 isoforms [48]; the long N- and C-terminal tails of NtNIPs, important for protein regulation, trafficking, and protein-protein interactions [73]; the distinctly short N-terminal of SIPs associated with their intracellular destination into the ER [74]; the long Loop C of NtXIPs, characteristically enriched with flexible glycine residues allowing it to tuck into the channel opening and interact with selectivity filter residues and permeating solutes [54, 75].

### NtAQP subcellular localisation

Determining AQP subcellular localisations can help elucidate physiological roles within the plant. For instance, integration into plasma membrane indicates solute transport in and out of the cell; localisation to the tonoplast implies a role in vacuole storage; or retained in the ER membranes to coordinate shuttling of substrates and nutrients between plant membranes [28, 56, 74, 76, 77]. We utilised sub-cellular localisation prediction software commonly used for fast in silico predictions of AQP isoform membrane integration. These software incorporate known sorting signals, amino acid composition and functional domains to generate results [55]. Using three software prediction tools (Plant-mPLoc, WolfPsort and YLoc) generally concluded that PIP, NIPs and XIPs are predominantly localise to the PM; all of the Plant-mPloc and some of the WolfPsort outputs predicted tonoplast localisation for the TIPs; and the SIP localisations were quite varied. Although these predictions are a useful beginning, it should be noted that we only found a 46% consensus in the predicted AQP subcellular localisations between the three software tools. The discrepancies highlight the complexity of AQP membrane integration processes and our current limited understanding of AQP trafficking motifs [69].

GFP:NtAQP fusions and crucially a set of established subcellular GFP marker lines, allowed us to directly visualise and confidently determine *in planta* sub-cellular localisation of representative NtAQPs. The representative PIP (*NtPIP2;5 t*), NIP (*NtNIP2*;1 s) and TIP (*NtTIP1;1 s*) NtAQPs had distinct sub-cellular localisations, consistent with what is known about these AQP subfamilies in other plants [28]. Concordant with studies of these subfamilies in other species [22, 78], we found that the NtPIP and NtTIP localised to the plasma membrane and tonoplast, respectively. The NtNIP2;1 co-localised to the PM and ER, which was not captured with the prediction software, which instead reported only PM integration. This sub-optimal PM targeting could limit the functional capacity of NtNIP2;1 and its subsequent physiological role (see discussion).

### Nicotiana AQP gene evolution

Tobacco recently descended from a allotetraploid hybridisation event between *N. sylvestris* and *N. tomentosiformis*, which are distantly related within the Nicotiana genus [79]. Genome downsizing is a widespread biological response to polyploidization, eventually leading to diploidization [80]. However, due to the short evolutionary time frame since its inception (0.2 M years), tobacco has undergone a limited amount of genome downsizing. As a result, the NtAQP family is characteristically comprised of sister gene pairs, which we could assign to their given parental origins. Tobacco has lost only around 10% of it duplicated genes with no observed preferential gene loss from either parent [43]. Concordant with this estimation, 7 gene loss events (~ 8.6% of total inherited parental AQPs) were identified in tobacco, with 3 and 4 of these being redundant ortholog losses from the *N. sylvestris* and *N. tomentosiformis* genomes, respectively. According to our expression analysis, the NtAQP gene copies inherited from both *N. sylvestris* and *N. tomentosiformis* (‘s’ and ‘t’ genes, respectively), were overall equally expressed, which agrees with broader genomic studies on tobacco [43]. The redundancy of the homeologs presumably would allow for one of the sister genes to accumulate mutations without an immediate effect on fitness, most often leading to non-functionalisation (gene-loss), or in some instances sub-functionalisation or even neo-functionalisation. To this end, we observed instances where one AQP gene of a sister pair was consistently preferentially expressed throughout several plant organs (e.g. *PIP1;1 s, PIP1;3 t, SIP2;1 t and NIP5;1 s*); suggesting that the redundant lower-expressing sister gene could become non-functional over time. Alternatively, some sister genes showed distinct tissue-specific diversification, such as the *NtPIP2;5* gene pair, where the s- and t-genes were more highly expressed in the roots and leaves, respectively, and which maybe candidates for sub- or even neo-functionalisation.

We were able to identify several AQP gene gain and loss events between the parents since their divergence within the Nicotiana genus, ~ 15 Ma ago [64]. Both the *N. sylvestris* and *N.tomentosiformis* have a genome rich of repeat expansion (accumulation of transposable elements), making them nearly 3 times the size of that of tomato and potato (2.6 Gb vs. 0.9 Gb) [64, 81, 82]. Regardless of the discrepancy in genome size, there was close conservation of AQP ortholog numbers within these diploid Solanaceae species; with the PIPs and TIPs consistently the larger subfamilies. We saw a significant diversity in XIPs occurring in the Solanum (tomato and potato) and the Nicotiana species. This diversity manifested as discrepancies in isoform numbers between the species and as lower sequence identity; depicted in the phylogeny as a separation of tomato, potato and Nicotiana isoforms into distinct groups. XIPs are a more recently characterised AQP subfamily, with isoforms lacking in monocots and in Brassicaceae, and having a lower overall sequence identity compared to other AQP subfamilies [17]. The tomato and potato XIP are predominantly found clustered on a single chromosome, indicating that recent segmental gene duplications within tomato and potato likely explain the lack of direct gene orthology to tobacco XIPs [83].

### Gene expression analysis

The NtAQP transcriptome was found to be largely conserved with those of its parental species, consistent with it recent evolutionary emergence. We also noted that the expression profiles between *AQP* sister genes within tobacco, correlated better than the expression patterns of the orthologous AQP between the parental lines. Such improved homogeneity in expression patterns is a common outcome of hybridisation events as both genomes (e.g. the ‘s’ and ‘t’ AQPs genes) are now subjected to the same regulatory network [84, 85].

Within tobacco, our NtAQP gene expression analysis revealed a wide range of patterns across tissue types, consistent with the known diversity of AQP functions [4]. It revealed that some AQPs had high levels across numerous tissues throughout the plant (e.g. *PIP1;3 t* and *PIP1;5, TIP1;1* sister pairs), implicating involvement in broad spanning processes (e.g. substrate transport from roots to shoots to flowers), while others had highly organ specific expression (e.g. *TIP1;*3, *NIP4;1*, and *TIP3;1* sister genes, in roots, flowers and seed capsules, respectively). In general, the XIPs and majority of NIPs had lower overall expression levels, although there is the possibility that their expression might change in response to a specific stimulus, or that they are expressed at similar levels, but in very specific cell types making up a small population of the total tissue sampled for RNA-seq.

Tissue specific expression patterns can help towards assigning physiological roles for the NtAQPs. We observed general trends between the AQP subfamilies. The NtXIPs were observed to have low but ubiquitous expression throughout the plant and previously reported to permeate bulky solutes such as urea and boric acid, but not water. Little is known about XIP physiological roles, but their unique transport capacity and rapid evolutionary diversification, even just within the Solanaceae, implies a role in environmental adaptive responses.

The tobacco PIPs appeared to have more isoforms with leaf-specific expression compared to the other subfamilies. These are likely to be involved in roles typically reported for PIPs across plants species, including; leaf cell expansion, leaf movement, mediating water exiting the xylem, control of stomatal aperture and gas transport (e.g. CO_2_) for photosynthesis [86–88]. Several PIPs have targeted expression in flowers (*PIP1;7 t*, *PIP1;8 s*, *PIP2;2 t*, *PIP2;3 t*, and *PIP2;8*, *PIP2;9*, *PIP2;11, PIP2;13* sister pairs), some of which would be involved in mediating water supply during stigma, anther and petal development [89, 90].

Much like the PIPs, several isoforms within the NIPs (*NIP4;3 s* and *NIP4;1* and *NIP4;2* sister genes) and TIPs (*TIP5;1* sister genes) had targeted expression to the flower. The tissue-specificity of these *NtNIP*s and *NtTIP*s is consistent with the floral tissue localisation of Arabidopsis *NIP4;1*, *NIP4;2* and *TIP5;1*, which have known roles in pollen development and pollen germination [53, 91]. Additionally, we identified *NtTIP3;1* and *NtTIP3;2* as being exclusively expressed in the seed capsule. This is consistent with the seed-specific expression of their orthologs in other species [92–94] where they accumulate in mature embryos and later function in water uptake during seed imbibition and germination [94–96]. The consistent expression pattern between species implies functional conservation, meaning that *NtNIP4;1*, *NtNIP4;2* and *NtTIP5;1* likely fulfil roles in different aspects of tobacco pollen biology, and *NtTIP3;1* and *NtTIP3;2* are expected to aid tobacco seed germination.

Several PIP and TIP isoforms were found with exclusive or preferential expression in the roots (e.g. *PIP1;1*, *PIP2;4*, *PIP2;5 s*, *PIP2;6*, *TIP1;2*, *TIP1;3*, *TIP2;5* and TIP2;2 s), where they could be functioning in lateral root emergence [97, 98], regulation of cell water uptake and homeostasis [33], or nutrient absorption through ammonium loading in vacuoles [99, 100]. The latter possible role of ammonium loading is especially pertinent to the two NtTIP2 proteins listed, which have a histidine residue in the ar/R LC position characteristic of ammonia transporting TIPs [51] .

The putative roles put forward for the various NtAQPs above, could equally apply to many of the tomato and potato AQPs and vice versa, given the general family-wide conservation in tissue-specific expression patterns between these three Solanaceae species. The generally high conservation in expression patterns between Solanaceae AQP orthologs supports the accuracy of our NtAQP orthology; assigned based on protein sequence homology. The similarity at both the protein and transcript levels strongly implies functional conservation for many of the AQP orthologs across these Solanaceae species. Knowledge of the extent of such conservation is valuable as it can help facilitate translation of findings across Solanaceae species for traits of agronomic importance and help direct engineering efforts. Deviations are also interesting (of which we observed several), as they hint at potential novel species-specific functions, or help explain physiological differences between species. For example, NIP2;1 is an unique NIP with a distinct GSGR ar/R filter motif and a precise loop C spacing between NPA motifs allowing it to permeate and aid silicon transport from root to shoot in a number of high silicon accumulating species [101, 102]. But, Solanaceae species are considered poor silicon accumulators [101, 102], which matches an apparent deterioration of the *NIP2;1* lineage in Solanaceae as seen in our cross-species comparisons with; *NIP2;1* being lost in *N. tomentosiformis* prior to tobacco hybridisation; a subsequent absence of a *NtNIP2;1 t* gene; both N.sylNIP2;1 and NtNIP2;1 s have a unfavourable loop C length for silicon transport, as does SlNIP2;1; potato does not possess a *NIP2;1*; the different expression patterns of *NtNIP2;1* and *SlNIP2;1* hint at diverging roles; NtNIP2;1:GFP is poorly localised to the PM likely limiting function capacity; and no other NtNIP has a GSGR ar/R filter configuration for redundancy.

## Conclusions

We determined that the tobacco AQP family consists of 76 members divided into five subfamilies each with subtle characteristic variations in protein structures, pore lining residues, and post-translational regulatory mechanisms. Characterisations of key residues and regions broaden our knowledge of AQP biology by guiding future functional studies to help identify substrate specificity residue combinations. The annotation of putative post-translational regulatory sites supports current knowledge of AQP regulation not only within the more widely studied PIP subfamily, but also across the TIP, NIP, SIP and XIP sub-groups. Members of the different NtAQP subfamilies were found to localise to specific sub-cellular membranes, which contribute collectively to a dynamic and extensive transport system. These subcellular profiles help towards elucidating physiological roles with, for example, PM-localising NtAQPs likely facilitating diffusion of solutes in and out of cells, and tonoplast-localising isoforms helping with intracellular distribution of solutes. Tobacco is a recent allotetraploid, which accounts for its large AQP family size and characteristic phylogenetic pairing of sister genes inherited and retained from its parents; *Nicotiana sylvestris* and *Nicotiana tomentosiformis.* By establishing heritage of NtAQP sister genes we were able to reconstruct the recent evolutionary history of the NtAQP family, which contributes to establishing potential functional homology of candidate genes. Expression analysis of the NtAQPs revealed diverse tissue-specificities, consistent with the broad spanning physiological functions of AQP. Some NtAQPs were expressed widely, while other showed specialised or strong preferential expression within a single tissue. We found that the expression specificity for a number of NtAQPs resembled that of orthologous AQPs with established physiological roles in other species, allowing us to assign putative homologous functions in tobacco. The conservation in AQP protein structure and gene expression patterns were high with other Solanaceae species, which will facilitate the translation of knowledge from tobacco into closely related and horticulturally important crops.

## Methods

### Identification of tobacco, *N.sylvestris* and *N.tomentosiformis* AQPs

The tobacco genome and the protein sequences for TN90 [42] and K326-Nitab4.5v [43] cultivars were obtained from the Solanaceae Genomics Network [103] and imported into the Geneious (V9.1.5) software [104]. To comprehensively identify putative aquaporin genes in tobacco, multiple BLASTP searches were performed against the TN90 tobacco predicted proteome, using each of the potato (*Solanum tuberosum*) and tomato (*Solanum lycopersicum*) aquaporin proteins sequences as queries. From each individual homology search, the top 3–5 matches were compiled as putative NtAQPs; with the list being consolidated at the end of the search routine. A similar process was used to identify AQPs in *N. sylvestris* and *N. tomentosiformis* (tobacco parental genomes), however tobacco aquaporin coding sequences were used in BLASTN queries. Sequence alignments were conducted using MUSCLE [105]. Whole family and sub-family sequence alignments were used to flag aberrant AQP protein sequences for closer inspection.

### Phylogenetic analysis and classification of tobacco, *N. sylvestris* and *N. tomentosiformis* AQPs

MUSCLE aligned nucleotide or protein sequences were used to construct phylogenetic trees using neighbour-joining (NJ) method (pair-wise deletion; bootstrap = 1000) in MEGA7 software [106]. Tobacco AQP naming convention was based on homology to that of the tomato AQPs. *N. sylvestris* and *N.tomentosiformis* AQP gene names were assigned based on homology to tobacco AQPs.

### Structural features of tobacco AQPs

The tobacco aquaporin intron/exon structures were identified by aligning CDS and genomic sequences. Comparisons of gene sequences (computed and our curations) and RNA-seq data were visualised through JBrowse. The topologies of the curated NtAQPs were defined using TOPCONS [107]. The complement of known functionally relevant residues were collected from MUSCLE aligned NtAQP protein sequences. Alignment statistics (e.g. % sequence identity and similarity using BLSM62 matrix) were collected from MUSCLE aligned sequences of individual subfamilies. Prediction of phosphorylation sites were performed using NetPhos 3.1 prediction score ≥ 0.8 [108].

Subcellular localisation predictions were achieved using; YLoc [55], Wolf PSort [109] and Plant-mPloc [110].

### Subcellular localisation *in planta* (Arabidopsis)

Tobacco AQP GFP fusion constructs were generated via Gateway cloning of a TIP (*NtTIP1,1 s*), PIP (*NtPIP2;5 t*) and NIP (*NtNIP5;1 t*) coding sequences from pZeo entry vectors into the pMDC43 destination vector [111]; which produced N-terminal GFP:NtAQP fusion proteins driven by the constitutive 2x35S CaMV promoter. Arabidopsis transgenic lines were generated via agrobacterium (GV3101) floral dipping plant transformation method (Clough and Bent 1998). The GFP marker line (MG0100.15) used as a cytosolic localisation marker was generated in our lab via the Gateway cloning of the mGFP6 variant of GFP contained as a pZeo entry clone into the pMDC32 destination vector [111]; which drives constitutive expression of the mGFP6 transgene via the 2x35S CaMV promoter. The PM:GFP line was also generated in our lab, built in the pMDC83 Gateway destination vector and consisting of the Arabidopsis PIP2;1 (an already established PM marker [63]) with a mGFP6 C-terminal fusion, all driven by the 2x35S CaMV promoter.

Arabidopsis seeds were liquid sterilised using hypochlorite, washed several times and sown on Gamorg’s B5 medium containing 0.8% Agar and the antibiotic hygromycin for selection of transformants. After 8 days of growth, arabidopsis seedlings were gently removed from the agar, mounted in Phosphate Buffer (100 mM NaPO_4_ buffer, *pH* 7.2) on a standard slide and covered with coverslip, and visualised with a Zeiss LSM 780 Confocal microscope using a 40x water immersion objective (1.2 NA). Light micrographs of cortical cells in the root elongation zone were visualised using Differential Interference Contrast (DIC), with GFP fluorescence captured using excitation at 488 nm and emission detection across the 490–526 nm range. Autofluorescence was detected in the 570–674 nm range and excluded from GFP detection channel. Images were processed using Fiji (ImageJ) program [112].

### AQP gene expression analysis

Transcript expression of the identified aquaporins was extracted from published, publicly available datasets, via two avenues [1]; mining of processed transcript expression matrices and [2] analyses of raw RNA-Seq reads uploaded to GenBank Sequence Read Archive (SRA). Processed transcript expression of *N. tabacum* K326 [43] was extracted from The Sol Genomics Network [103]. Data was extracted as transcripts per million (TPM) and so was mined without further processing. This data set contained tissue specific expression of the leaf and root. Raw RNA-Seq reads from both *N. tabacum* K326 and TN90 [42] were downloaded from the GenBank SRA (TN90: SRP029183; K326: SRP029184) via command line into paired end fastq files. Read libraries were tissue specific from either the leaf, root, young leaf, young flower, mature leaf, mature flower, senescent leaf, senescent flower or dry capsule. On average each tissue was represented by a RNA-seq library of ~ 110 million paired reads. The raw reads were processed using Trimmomatic [113] to remove adapter sequences. Processed reads were aligned to the *N. tabacum* genome, either the K326 [43] or TN90 [42], using the Quasi align mode within Salmon [114] invoking a k-mer length of 31, with relative abundance reported as transcripts per million (TPM). Mapping rates of the K326 and TN90 transcriptomes were between 73 and 78%, and 89–94%, respectively. Raw RNA-seq reads for the parental genomes of *N. sylvestris* and *N. tomentosiformis* were obtained from [64]. RNA-seq libraries were libraries were derived from root, leaf, and flower tissues, with an average of 265 million paired reads for each tissue type. Reads were processed as above and mapped to the *N. sylvestris* and *N. tomentosiformis* genomes obtained from [64].

Tomato and potato root, leaf and flower expression data was retrieved through the EMBL-EBI Expression Atlas, and originally published by [115] and [116].

## Supplementary information

**Additional file 1: Table S1.** Tobacco AQP pseudo genes. Table of sequences that encode for incomplete AQPs within the tobacco TN90 genome sequence (Sierro et al. 2014), that we have subsequently assigned as pseudo genes. Notes on trans-membrane domains were sourced from analysis using TOPCONs protein topology prediction software. **Table S2.** Extended information on the 76 tobacco aquaporins identified in this study. Provided are protein lengths, gene identifiers, gene structures, chromosome and/or scaffold locations in the TN90 [1] (Sierro et al. 2014) and K326 [2] (Edwards et al. 2017) cultivar genomes, comparison of whether the computed gene models derived from each study matched gene structures curated in this study (Y-yes or N-no) and NCBI accessions. *NtTIP2;5 s*, *NtNIP4;2 s* and *NtNIP4;3 t* genes were not identified in the K326 [2] cultivar’s genome. Also listed are the corresponding tomato and potato orthologs and their respective gene (inton/exon) structures. **Table S3.** Amended annotations of previously reported tomato, potato and tobacco AQPs. In analysing the NtAQP family, we identified misannotations in previously reported AQPs from tomato (*Solanum lycopersicum*), potato (*Solanum tuberosum*) and tobacco (*Nicotiana tabacum*). Provided is a brief description of the error. Corrected sequences can be found in Additional file 3.

**Additional file 2: Figure S1.** AQP subfamily alignments for genes with incorrect protein sequences reported in Edwards et al. (2017). In red is the Edwards et al. (2017) predicted protein sequence and in black is the curated protein sequence from this study. **Figure S2.** Alignment of regions surrounding Histidine 207 in NtAQP1 (NtPIP1;5 s). Partial regions of a protein sequence alignment surrounding Histidine 207 of the NtAQP1 (NtPIP1;5) identified in this study, against the seemingly erroneous NtAQP1 sequence reported in (Biela et al., 1999; NCBI AF024511 and AJ001416) and closest BlastP matches from various other Solanaceae species. **Figure S3.** Phylogeny of Arabidopsis, tomato, rubber tree, rice, soybean and tobacco AQPs. Figure too large for this PDF; See Additional file 4. **Figure S4.** Phylogeny of Arabidopsis and currently identified Solanaceae AQPs. Phylogenetic trees for each AQP sub-family were generated using the neighbour-joining method from MUSCLE aligned protein sequences. Confidence levels (%) of branch points generated through bootstrapping analysis (*n* = 1000). Solanaceae species included in this phylogeny include; *N.sylvestris* (orange), *N.tomentosiformis* (blue), tomato (green), potato (brown) and tobacco (black). Arabidopsis genes are coloured red. Black stars indicate NtAQPs which did not have an obvious tomato ortholog. **Figure S5.** Sequence alignment of C-terminal tails of NtPIP and NtNIP proteins. Serine residues in red are those predicted to be phosphorylated by NetPhos 3.1 (prediction score ≥ 0.8). Underlined red serine residues in GmNOD26, SoPIP2;1 and AtPIP2;1 have been experimentally confirmed as being phosphorylated in plants. Bold residues indicate the substitution of strongly conserved positively charged Lys(K)/Arg(R) residues to a His(H) residue (blue) occurring in NtPIP1;5 and NtPIP2;1 proteins. **Figure S6.** Comparisons of expression profile between AQPs from tobacco (*NtAQPs* and *NtAQPt*, genes), *Nicotiana sylvestris* (*N.syl*) and *Nicotiana tomentosiformis* (*N.tom*)*.* Correlations of relative transcript abundances was compared in two-dimensions; (i) between AQPs within a given tissue (vertically) and (ii) a given AQP across tissues (horizontally). **Figure S7.** Tissue-specific expression patterns of tomato XIP isoforms (*SlPXIP1;1-SlXIP1;6*) and the tobacco *NtXIP1;7* sister genes. Comparison of relative gene expression in roots, leaves and flowers of tobacco *NtXIP1;7* sister genes (blue) against all the tomato XIP isoforms (red, *SlXIP1;1-SlXIP6*), with potato orthologs (brown), in an attempt to find matches between the various XIPs which were difficult to assign orthology based on protein sequence alone.

**Additional file 3: **Repository of sequences examined in this study. Genomic (gff3 format), CDS, and protein sequences for all 76 NtAQPs. CDS and protein sequences for all *N.sylvestris* and *N.tomentosiformis* AQPs. Amended sequences for potato StXIP3;1, StXIP4;1, and tomato SlXIP1;6, SlPIP2;1, SlTIP2;2 proteins (see also Additional file 1: Table S3)

**Additional file 4: Figure S3.** Phylogeny of Arabidopsis, tomato, rubber tree, rice, soybean and tobacco AQPs. Phylogenetic analysis of tobacco AQPs with those from species belonging to a diverse set of plant species from across the angiosperm lineage: Arabidopsis (Brassicales), tomato (Solanales), rubber tree (Malpighiales), rice (Poales) and soy bean (Fabales). Tree was generated using the neighbour-joining method from MUSCLE-aligned protein sequences. Confidence levels (%) of branch points generated through bootstrapping analysis (*n* = 1000). AQP subfamilies annotated are TIP (blue), NIP (purple), XIP (yellow), PIP (orange), SIP (green).

## Data Availability

We declare that the dataset(s) supporting the conclusions of this article are included within the article and its additional file(s). All of our curated aquaporin CDS nucleotide and protein sequence data reported for *Nicotiana tabacum*, *N. sylvestris* and *N. tomentosiformis* is available in the Third-Party Annotation Section of the DDBJ/ENA/GenBank databases under the accession numbers TPA: BK011376-BK011532; BankIt2254789.

## Abbreviations

AQP: Aquaporin
ar/R: Aromatic/Arginine
CDS: Coding DNA sequence
ER: Endoplasmic reticulum
MIP: Major intrinsic protein
NIP: Nodulin26-like Intrinsic protein
NPA: Asparagine-proline-alanine
N. syl: *Nicotiana sylvestris*
N. tom: *Nicotiana tomentosiformis*
Nt: *Nicotiana tabacum*
PIP: Plasma membrane intrinsic PROTEIN
PM: Plasma membrane
Sl: *Solanum lycopersicum*
St: *Solanum tuberosum*
TIP: Tonoplast intrinsic protein
TM: Transmembrane domain
Tono: Tonoplast
XIP: X Intrinsic Protein
