# Supplementary material for: Genome-wide identification and characterisation of Aquaporins in Nicotiana tabacum and their relationships with other Solanaceae species
Source: BMC Plant Biol. 2020 Jun 9;20:266. doi: 10.1186/s12870-020-02412-5 (PMC7285608; doi:10.1186/s12870-020-02412-5)
Supplement: Supplementary file 2 — Additional file 2: Figure S1. AQP subfamily alignments for genes with incorrect protein sequences reported in Edwards et al. (2017). In red is the Edwards et al. (2017) predicted protein sequence and in black is the curated protein sequence from this study. Figure S2. Alignment of regions surrounding Histidine 207 in NtAQP1 (NtPIP1;5 s). Partial regions of a protein sequence alignment surrounding Histidine 207 of the NtAQP1 (NtPIP1;5) identified in this study, against the seemingly erroneous NtAQP1 sequence reported in (Biela et al., 1999; NCBI AF024511 and AJ001416) and closest BlastP matches from various other Solanaceae species. Figure S3. Phylogeny of Arabidopsis, tomato, rubber tree, rice, soybean and tobacco AQPs. Figure too large for this PDF; See Additional file 4. Figure S4. Phylogeny of Arabidopsis and currently identified Solanaceae AQPs. Phylogenetic trees for each AQP sub-family were generated using the neighbour-joining method from MUSCLE aligned protein sequences. Confidence levels (%) of branch points generated through bootstrapping analysis (n = 1000). Solanaceae species included in this phylogeny include; N.sylvestris (orange), N.tomentosiformis (blue), tomato (green), potato (brown) and tobacco (black). Arabidopsis genes are coloured red. Black stars indicate NtAQPs which did not have an obvious tomato ortholog. Figure S5. Sequence alignment of C-terminal tails of NtPIP and NtNIP proteins. Serine residues in red are those predicted to be phosphorylated by NetPhos 3.1 (prediction score ≥ 0.8). Underlined red serine residues in GmNOD26, SoPIP2;1 and AtPIP2;1 have been experimentally confirmed as being phosphorylated in plants. Bold residues indicate the substitution of strongly conserved positively charged Lys(K)/Arg(R) residues to a His(H) residue (blue) occurring in NtPIP1;5 and NtPIP2;1 proteins. Figure S6. Comparisons of expression profile between AQPs from tobacco (NtAQPs and NtAQPt, genes), Nicotiana sylvestris (N.syl) and Nicotiana tomentosif [file 12870_2020_2412_MOESM2_ESM.pdf]

Additional File 2: Supplementary Figures

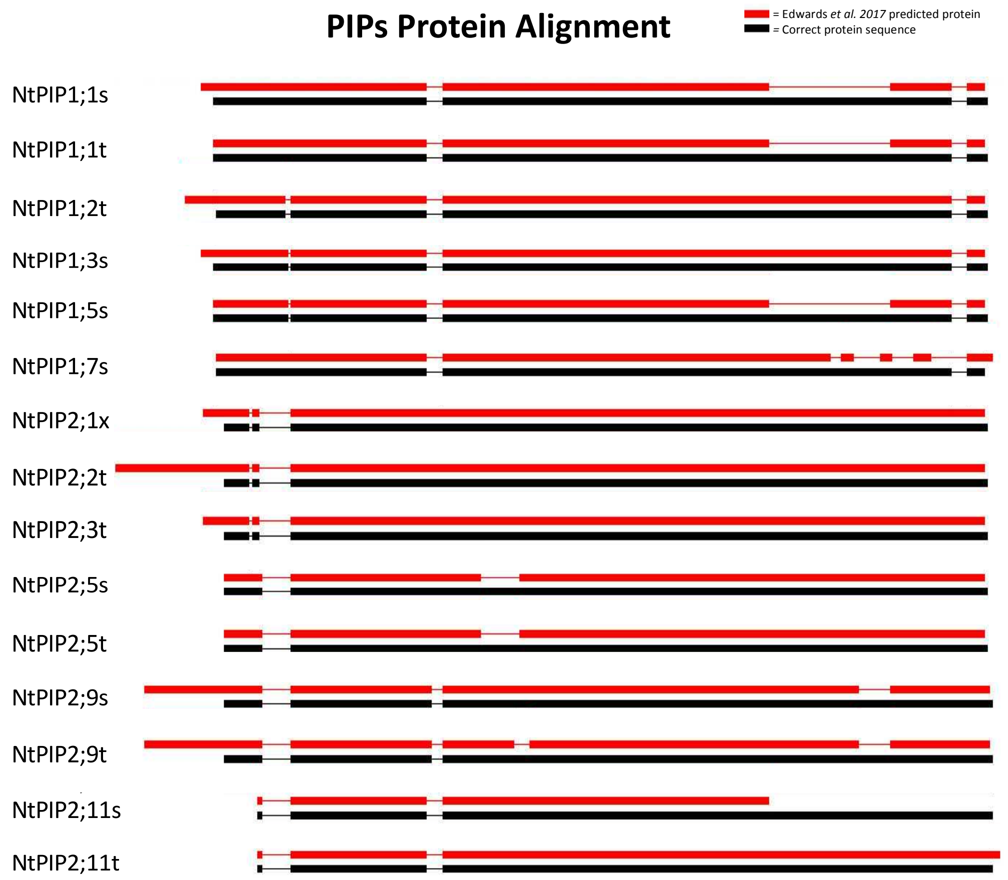

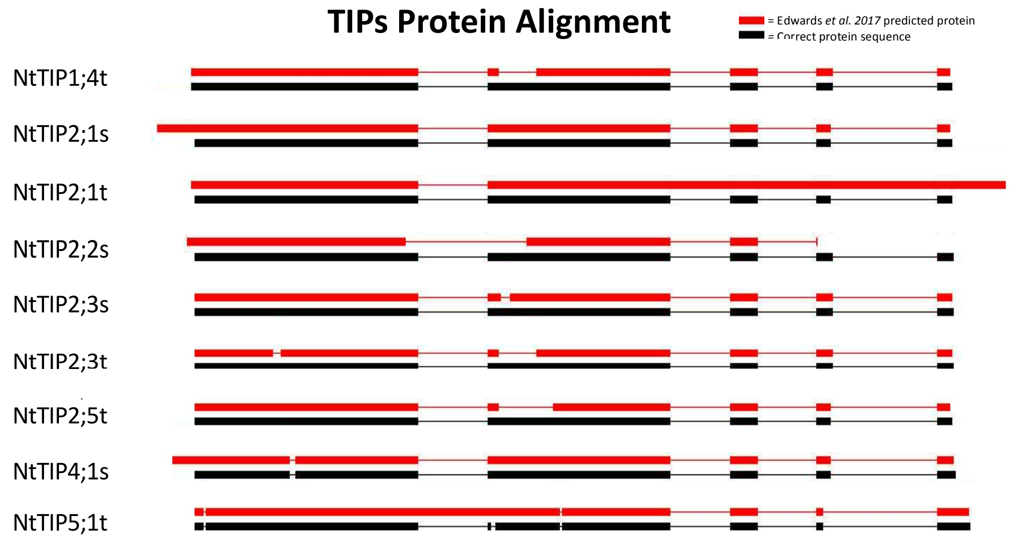

Figure S1. AQP subfamily alignments for genes with incorrect protein sequences reported in Edwards *et al.* (2017). In red is the Edwards *et al.* (2017) predicted protein sequence and in black is the curated protein sequence from this study.

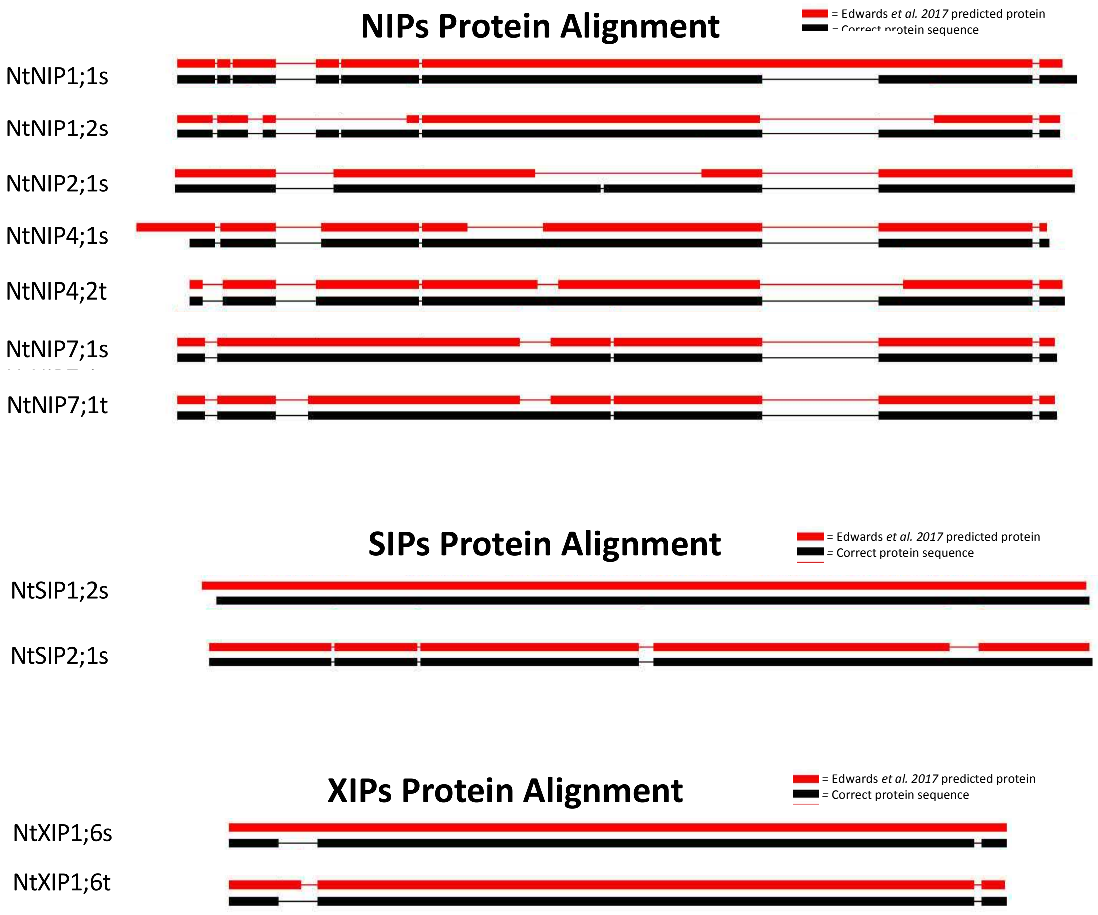

Figure S1. ***Continued***

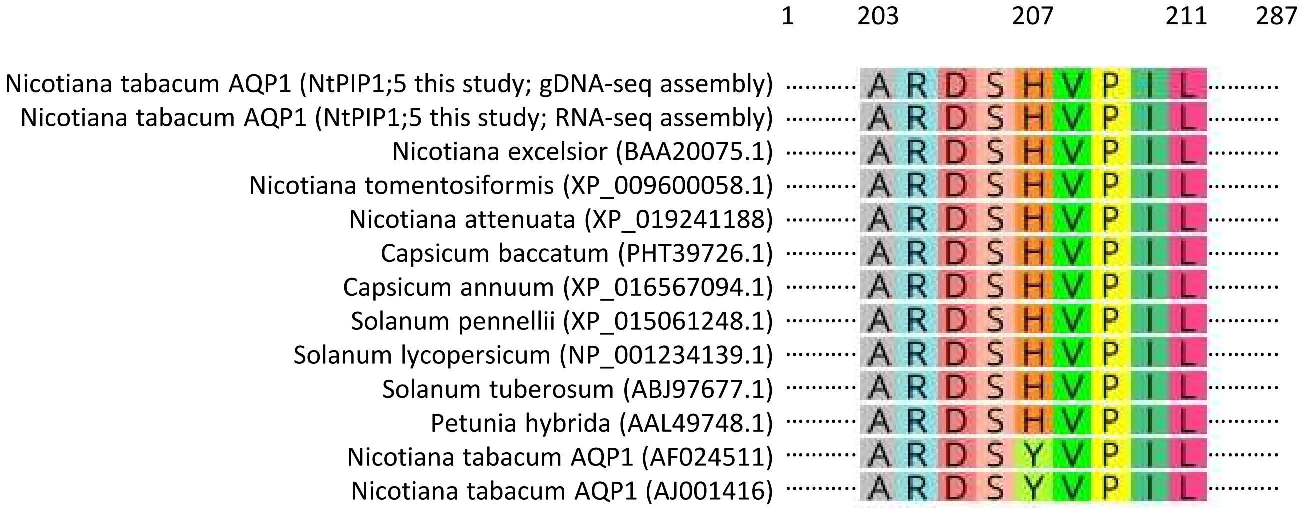

Figure S2. Alignment of regions surrounding Histidine 207 in NtAQP1 (NtPIP1;5s). Partial regions of a protein sequence alignment surrounding Histidine 207 of the NtAQP1 (NtPIP1;5) identified in this study, against the seemingly erroneous NtAQP1 sequence reported in (Biela et al., 1999; NCBI AF024511 and AJ001416) and closest BlastP matches from various other Solanaceae species. The H207 for NtAQP1 (NtPIP1;5) was identified using multiple independent sources, including both gDNA-seq assemblies and RNA-seq mapped reads from both TN90 and K326 cultivars (Sierro et al., 2014; Edwards et al., 2017).

Figure S3. Phylogeny of Arabidopsis, tomato, rubber tree, rice, soybean and tobacco AQPs . Phylogenetic analysis of tobacco AQPs with those from species belonging to a diverse set of plant species from across the angiosperm lineage: Arabidopsis (Brassicales), tomato (Solanales), rubber tree (Malpighiales), rice (Poales) and soy bean (Fabales). Tree was generated using the neighbour-joining method from MUSCLE-aligned protein sequences. Confidence levels (%) of branch points generated through bootstrapping analysis (n=1000). AQP subfamilies annotated are TIP (blue), NIP (purple), XIP (yellow), PIP (orange), SIP (green).
NEW FIGURE LEGEND

Figure too large for this PDF; See Additional file 4

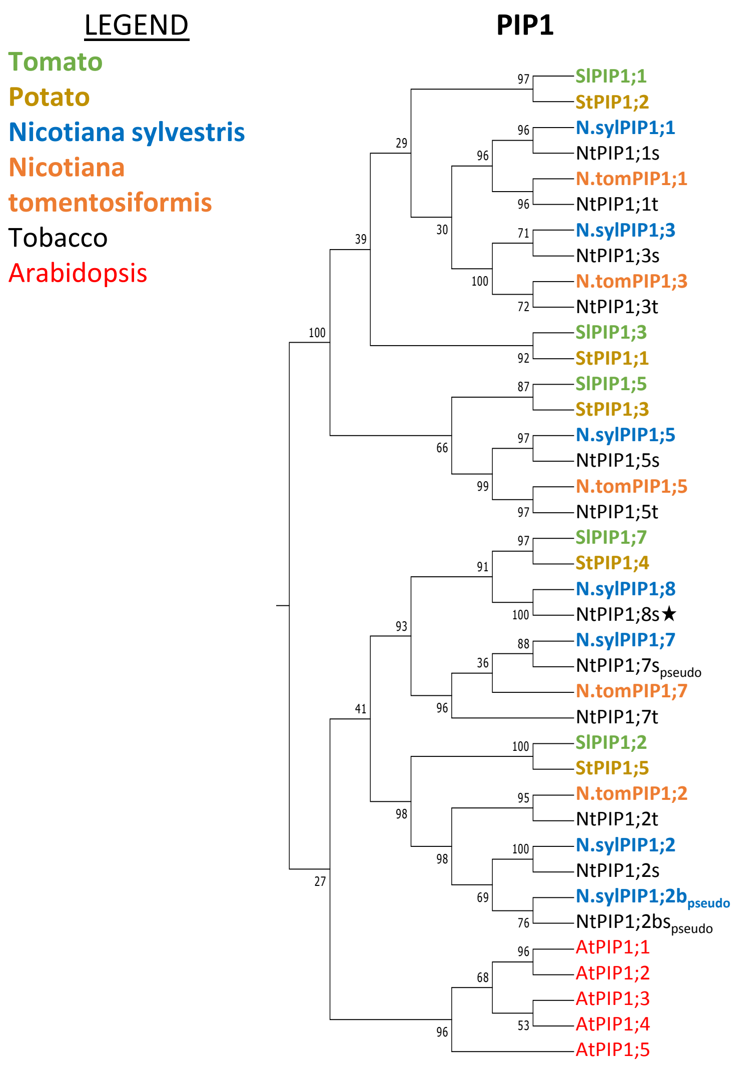

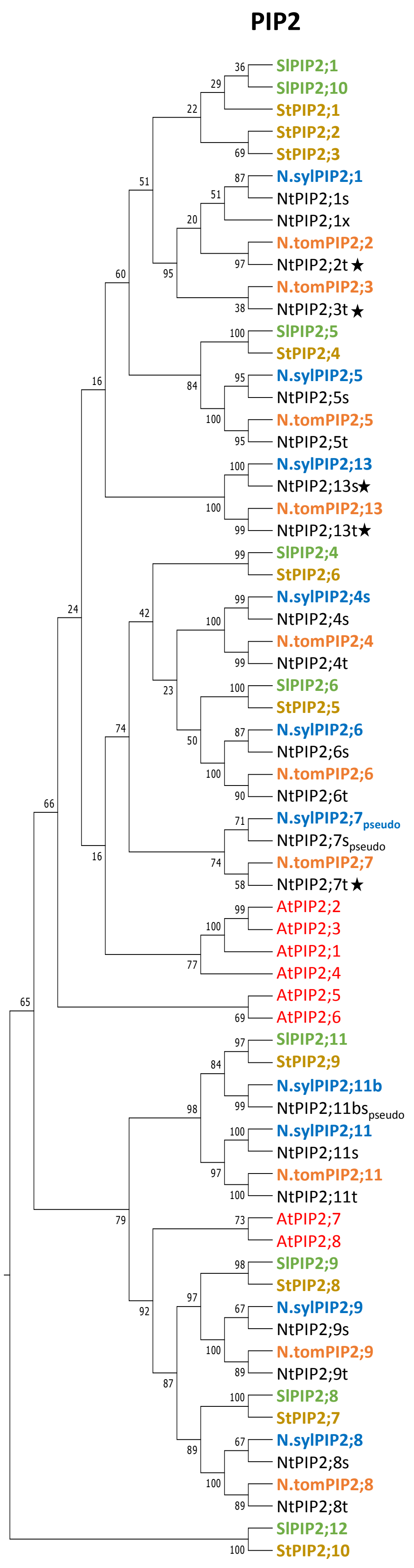

Figure S4. Phylogeny of Arabidopsis and currently identified Solanaceae AQPs. Phylogenetic trees for each AQP sub-family were generated using the neighbour-joining method from MUSCLE aligned protein sequences. Confidence levels (%) of branch points generated through bootstrapping analysis (n=1000). Solanaceae species included in this phylogeny include; *N.sylvestris* (orange), *N.tomentosiformis* (blue), tomato (green), potato (brown) and tobacco (black). Arabidopsis genes are coloured red. Black stars indicate NtAQPs which did not have an obvious tomato ortholog.

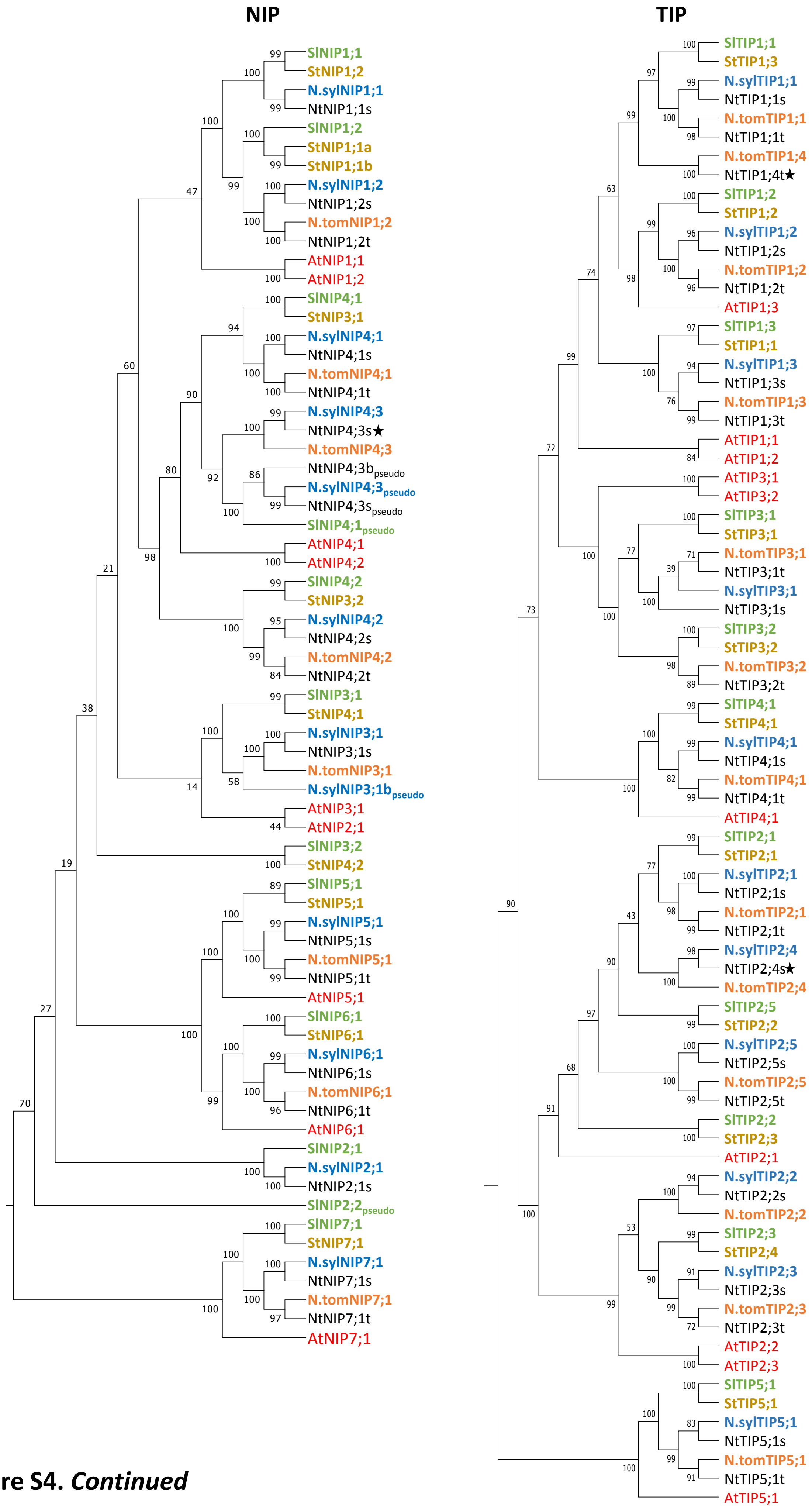

Figure S4. ***Continued***

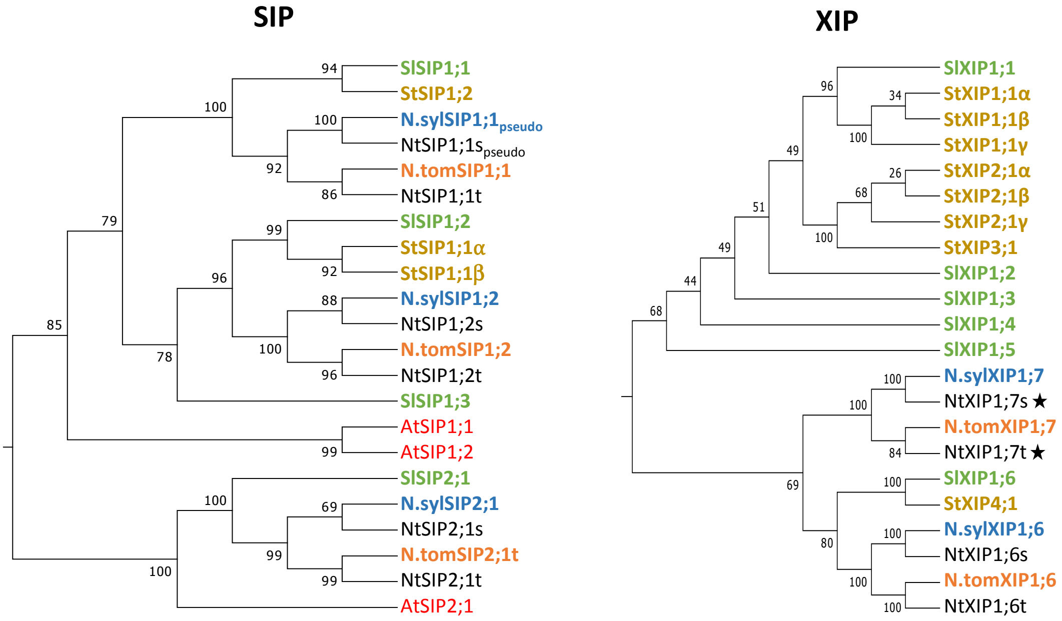

Figure S4. ***Continued***

NIP C-terminal tail

```
>GmNOD26   IVRYTDKPLSETTKSASF---LKGRAASK*----------------------
>NtNIP2;1s FIRVTDKPVHAIAPGQSFSFKLRRMKSNDEEQGV*-----------------
>NtNIP4;3s LIRFTNKPLLQLVKSRSF---LPKLRE*------------------------
>NtNIP4;1s LIRSTDKPLRELAKSASS---LRS*---------------------------
>NtNIP4;1t LIRSTDKPLRELAKTASS---LRS*---------------------------
>NtNIP4;2s LIRFTEKPLRELTKSSTF---LKSMSRSHT*---------------------
>NtNIP4;2t LIRFTEKPLKELTKSSTF---LKSMSRSHA*---------------------
>NtNIP1;1s IIRFTDKPLREITKSGSF---LKSKISNT-----------------------
>NtNIP1;2s IIRFTDKPLREITKSGSF---LKSIRSSKSLRSST*--------------
>NtNIP1;2t IIRFTDKPLREITKSGSF---LKSIRSST-----------------------
>NtNIP7;1s LLRLQGWSCKPNSTPTTT---HQHNPL*------------------------
>NtNIP7;1t LLRLQGWSCKPNSTPTTT---HQHNPL*------------------------
>NtNIP5;1s LVKLRGDDSSETPRQ------VRSFRR*------------------------
>NtNIP5;1t LVKLRGDDSTETPRQ------VRSFRR*------------------------
>NtNIP6;1s AVKLPNEDDNNHGKPSV----EHSFRR*------------------------
>NtNIP6;1t AVKLPNEDDNNHGKPSL----EHSFRR*------------------------
>NtNIP3;1s LMRLTNKSWGEAVKEISESQKVIEVSSKDKVICKCS...50...RSYKMYI*
```

PIP C-terminal tail

```
>SoPIP2;1   LRAAAIKALGSFRSNPTN*
>AtPIP2;1   LRASGSKSLGSFRSAANV*
>NtPIP1;5s  IRAIP------FHKSS*--
>NtPIP1;5t  IRAIP------FHKSS*--
>NtPIP1;3s  IRAIP------FKSKA*--
>NtPIP1;3t  IRAIP------FKSKA*--
>NtPIP1;1s  IRAIP------FKSKS*--
>NtPIP1;1t  IRAIP------FKSKS*--
>NtPIP1;7s  IRAIP------FKSK*---
>NtPIP1;7t  IRAIP------FKSK*---
>NtPIP1;2s  IRAIP------FKSGNLA*
>NtPIP1;2t  IRAIP------FRSGN*--
>NtPIP2;11s LRAQAAKTLSSFHSNPSI*
>NtPIP2;11t LRAQAAKTLSSFHSNSSI*
>NtPIP2;13s LRAGAAKALGSFRSSSQV*
>NtPIP2;13t LRAGAAKALGSFRSSSQV*
>NtPIP2;5s  LRAGAVKALGSFRSNA*--
>NtPIP2;5t  LRAGAVKALGSFRSNA*--
>NtPIP2;3t  LRAGAVKALGSFRSNA*--
>NtPIP2;2t  LRAGAVKALGSFRSNA*--
>NtPIP2;1s  LRAGAVKALGSFRSNA*--
>NtPIP2;1x  LRAGAVKALGSFRSNA*--
>NtPIP2;7t  LRAGAVKALGSFRSNA*--
>NtPIP2;6s  LRAGALKALGSFRSNA*--
>NtPIP2;6t  LRAGALKALGSFRSNA*--
>NtPIP2;4s  LRAGAIKALGSFRSNA*--
>NtPIP2;4t  LRAGAIKALGSFRSNA*--
>NtPIP2;9s  LRAGAVKALGSFRSNPTN*
>NtPIP2;9t  LRAGAVKALGSFRSNPTN*
>NtPIP2;8s  LRGSAIKALGSFRSNPTN*
>NtPIP2;8t  LRGSAIKALGSFRSNPTN*
```

Figure S5. Sequence alignment of C-terminal tails of NtPIP and NtNIP proteins. Serine residues in red are those predicted to be phosphorylated by NetPhos 3.1 (prediction score ≥ 0.8). Underlined red serine residues in GmNOD26, SoPIP2;1 and AtPIP2;1 have been experimentally confirmed as being phosphorylated in plants. Bold residues indicate the substitution of strongly conserved positively charged Lys(K)/Arg(R) residues to a His(H) residue (blue) occurring in NtPIP1;5 and NtPIP2;1 proteins.

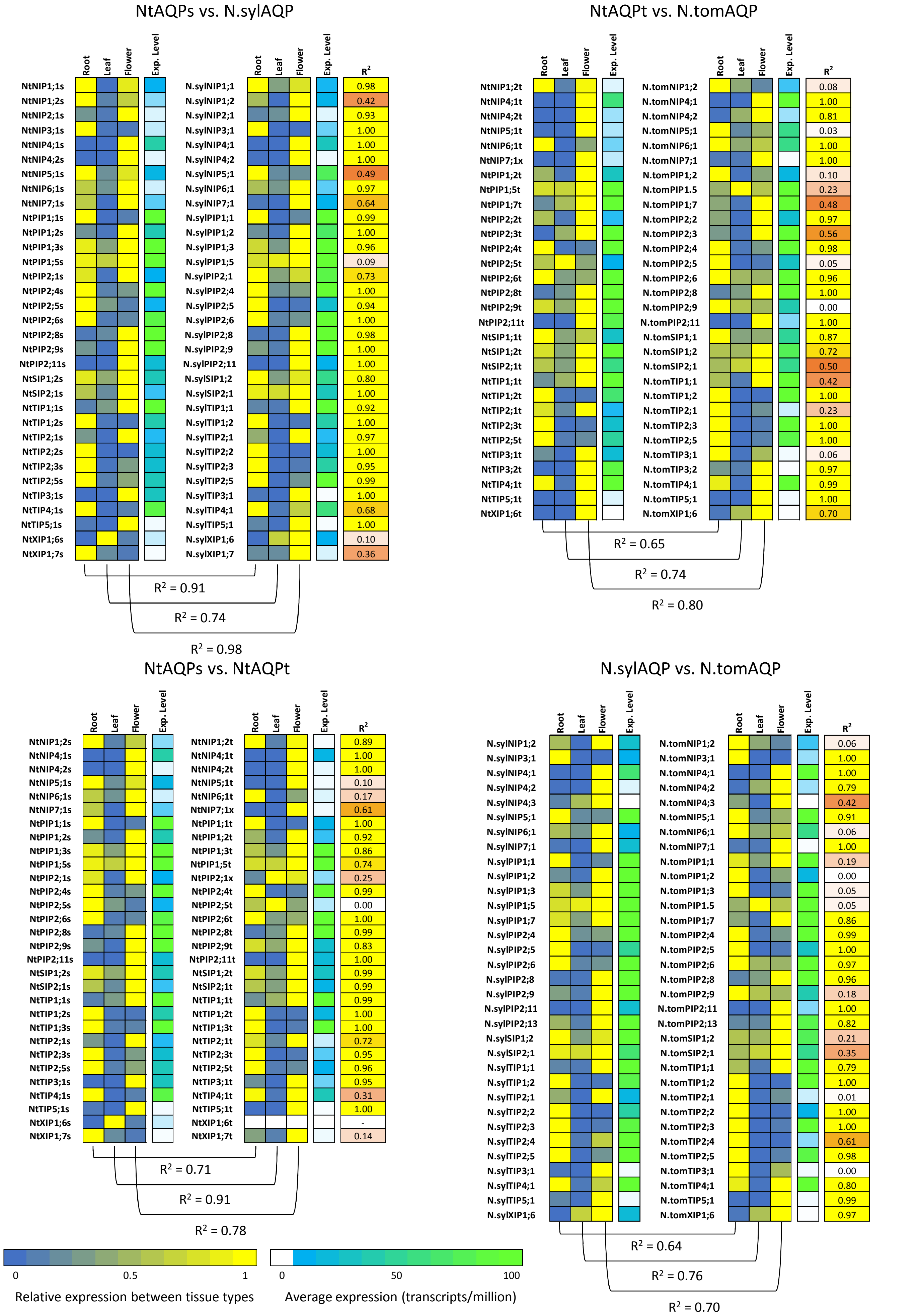

Figure S6. Comparisons of expression profile between AQPs from tobacco (*NtAQPs* and *NtAQPt*, genes), *Nicotiana sylvestris* (*N.syl*) and *Nicotiana tomentosiformis* (*N.tom*). Correlations of relative transcript abundances was compared in two-dimensions; (i) between AQPs within a given tissue (vertically) and (ii) a given AQP across tissues (horizontally).

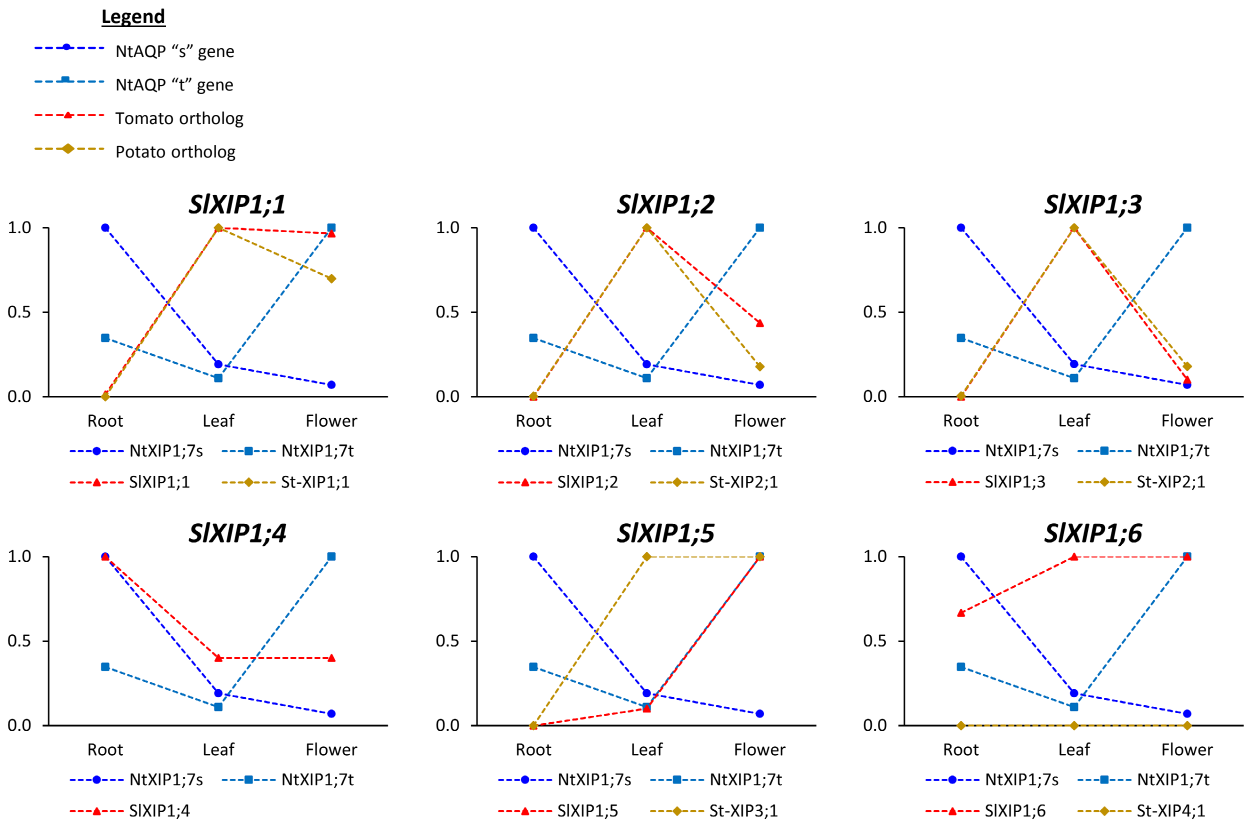

Figure S7. Tissue-specific expression patterns of tomato XIP isoforms (*SlPXIP1;1-SlXIP1;6*) and the tobacco *NtXIP1;7* sister genes. Comparison of relative gene expression in roots, leaves and flowers of tobacco *NtXIP1;7* sister genes (blue) against all the tomato XIP isoforms (red, *SlXIP1;1-SlXIP6*), with potato orthologs (brown), in an attempt to find matches between the various XIPs which were difficult to assign orthology based on protein sequence alone.
