# Supplementary figures and images for: Genome-wide identification and characterisation of Aquaporins in Nicotiana tabacum and their relationships with other Solanaceae species
Source: BMC Plant Biol. 2020 Jun 9;20:266. doi: 10.1186/s12870-020-02412-5 (PMC7285608; doi:10.1186/s12870-020-02412-5)

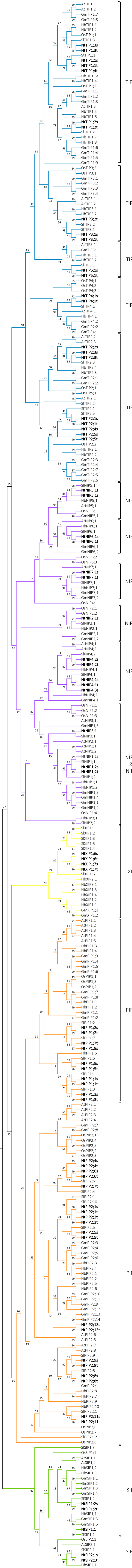

Supplement: Supplementary file 4 — Additional file 4: Figure S3. Phylogeny of Arabidopsis, tomato, rubber tree, rice, soybean and tobacco AQPs. Phylogenetic analysis of tobacco AQPs with those from species belonging to a diverse set of plant species from across the angiosperm lineage: Arabidopsis (Brassicales), tomato (Solanales), rubber tree (Malpighiales), rice (Poales) and soy bean (Fabales). Tree was generated using the neighbour-joining method from MUSCLE-aligned protein sequences. Confidence levels (%) of branch points generated through bootstrapping analysis (n = 1000). AQP subfamilies annotated are TIP (blue), NIP (purple), XIP (yellow), PIP (orange), SIP (green). [file 12870_2020_2412_MOESM4_ESM.pdf]
